# Huanglianjiedu Decoction as an effective treatment for oral squamous cell carcinoma based on network pharmacology and experimental validation

**DOI:** 10.1186/s12935-021-02201-6

**Published:** 2021-10-21

**Authors:** Lejun Zhang, Zhaoting Ling, Zhengqiang Hu, Guanmin Meng, Xinqiang Zhu, Huifang Tang

**Affiliations:** 1 Central Laboratory, The Fourth Affiliated Hospital, Zhejiang University School of Medicine, Yiwu, Zhejiang China; 2 Department of Pharmacology, School of Basic Medical Sciences, Zhejiang University, Hangzhou, Zhejiang China; 3 Department of Pharmacy, The Fourth Affiliated Hospital, Zhejiang University School of Medicine, Yiwu, Zhejiang China; 4 Department of Biochemistry, University of Alberta, Edmonton, AB Canada; 5 Department of Clinical Laboratory, Tongde Hospital of Zhejiang Province, Hangzhou, Zhejiang China

**Keywords:** Network pharmacology, Huanglianjiedu Decoction, Oral squamous cell carcinoma

## Abstract

**Background:**

Oral squamous cell carcinoma (OSCC) is one of malignant tumors in oral and maxillofacial region with high fatality. Huanglianjiedu Decoction (HLJDD) is a well-known traditional Chinese medicinal prescription, which consists of *Coptis chinensis Franch*, *Scutellaria baicalensis Georgi*, *Phellodendron amurense Rupr* and *Gardenia jasminoides J.Ellis*. Some clinical studies showed HLJDD had good effectiveness on OSCC, but the mechanism is unclear.

**Methods:**

In this study, potential components of HLJDD and putative targets were screened by Traditional Chinese Medicine Systems Pharmacology Database and Analysis Platform (TCMSP). Combining with potential targets of OSCC searched from Therapeutic Target Database (TTD) and Online Mendelian Inheritance in Man (OMIM), we drew protein–protein interaction (PPI) network by Cytoscape v3.2.0 software. After topological analysis we got core targets and further did Gene Ontology (GO) enrichment and Kyoto Encyclopedia of Genes and Genomes (KEGG) pathway analysis. Then we did the in vitro experiments to verify the major biological processes (cell cycle, apoptosis and proliferation) and signaling pathways (mitogen-activated protein kinase (MAPK), nuclear factor-kappa B (NF-κB), protein kinase B (AKT)) on OSCC cell lines, SCC-25 and CAL-27.

**Results:**

The potential component targets number of *Coptis chinensis Franch*, *Scutellaria baicalensis Georgi*, *Phellodendron amurense Rupr* and *Gardenia jasminoides J.Ellis* were 39, 93, 81and 88, respectively. Then we got 52 core targets which enriched in cell cycle, apoptosis, proliferation, MAPK activation etc. and obtained TOP30 pathways. On SCC-25 and CAL-27, HLJDD suppressed cell proliferation, induced late apoptosis and inhibited cell invasion and migration which were consistent with the results from network pharmacology analysis. Additionally, in cell cycle, we confirmed HLJDD inhibited G1 phase and arrested in S phase to reduce cell proliferation on SCC-25. In signaling pathways, HLJDD inhibited the phosphorylation of extracellular regulatory protein kinase 1/2 (ERK1/2) and NF-κB p65 (S468) on SCC-25 and CAL-27.

**Conclusions:**

HLJDD played a potential therapeutic role on OSCC via inhibiting p-ERK1/2 and p-NF-κB p65 (S468).

**Graphical abstract:**

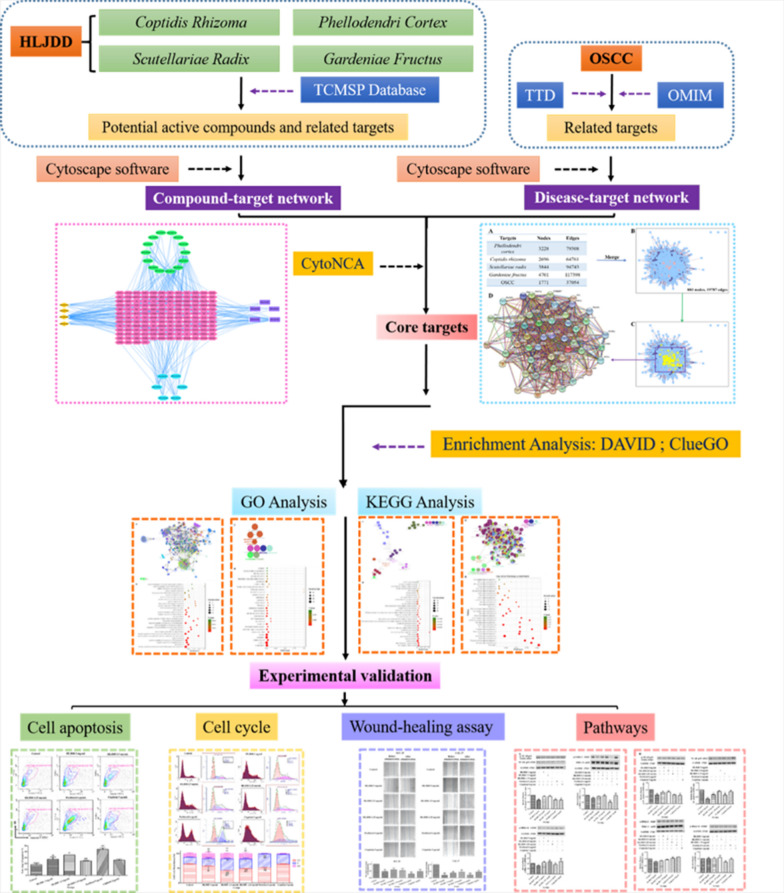

**Supplementary Information:**

The online version contains supplementary material available at 10.1186/s12935-021-02201-6.

## Background

Oral squamous cell carcinoma (OSCC) is one of squamous cell carcinoma in the oral mucosal epithelium. Nearly 300,000 patients especially among young people worldwide have suffered OSCC and the prevalence is still increasing [1]. Many patients were found at the advanced stage, resulting in a high mortality rate of this disease [2]. Data showed 5-year survival rate of OSCC is only 50% [3, 4], so finding the effective therapy for OSCC is urgent. Following the recognition of biomarkers, molecular-targeted therapy offered new approach for cancer therapy [5], including OSCC therapy. The key molecules and pathways for targeted therapy include growth factor receptors, Mitogen-activated protein kinase/Extracellular regulated protein kinases (MAPK/ERK) pathways, angiogenesis and epithelial-mesenchymal transition [6], which provide the possibility for individualized treatments.

Network pharmacology was firstly proposed and systematically described by Andrew L Hopkins in the *Nature Biotechnology* [7]. Based on nodes of the network to design multi-target drugs and synergistic effects of targeted drugs, network pharmacology is widely used to explore the effects or mechanisms of drugs on many diseases through an overall systemic perspective [8], such as respiratory diseases (acute lung injury, lung cancer etc.) [9, 10], kidney disease like pediatric adrenocortical carcinoma [11], liver disease like hepatocellular carcinoma [12] etc. Traditional Chinese Medicine (TCM) with a history of more than 5000 years, has a variety of herbal formulas. However, there are too many components to analyze the main mechanism of herbal formulas [13]. Many studies used network pharmacology to precisely analyze the targets of multiple drug components based on big databases, providing a novel analysis approach to screen the active ingredients and targets of TCM [8]. An new model called "multi-drug, multi-target" derived from network pharmacology, provides a new method to analyze and predict the targets and mechanisms of TCM [14].

Huanglianjiedu Decoction (HLJDD) is a classic ancient prescription of Qingrejiedu Decoction, which consists of *Coptis chinensis Franch*, *Scutellaria baicalensis Georgi*, *Phellodendron amurense Rupr* and *Gardenia jasminoides J.Ellis* [15]. The main effects of HLJDD are "Purging the fire and detoxifying". Previous researches suggested that HLJDD had therapeutic potentials on Alzheimer's disease [16], stroke [17], gastritis [18]. Growing evidences furtherly demonstrated that HLJDD had anti-infective [19], anti-inflammatory and anti-allergic effects [20], and so on. Moreover, HLJDD showed anti-tumor activities both in vivo and in vitro, it could significantly inhibit the growth of experimental tumor H22 in mice in a dose-dependent manner, and the drug serum had obvious inhibitory effects against human cancer cells, such as Swille, SPC-A-1, SGC-7901 and MCF-7 [21]. In clinical research, the effective rate of HLJDD for tongue cancer was nearly 83.3% [22]. All those evidences demonstrated that HLJDD was a very promising TCM prescription in cancer therapy. However, the mechanism of HLJDD on cancer is rarely. Wang et al. suggested that HLJDD inhibited eEF2 to block the progression of hepatocellular carcinoma on experimental animal model [23]. But the detailed mechanisms are still unclear. Based on the high cure rate of HLJDD on tongue cancer, we designed this study to explore the mechanisms of HLJDD on OSCC.

In this study, firstly we used network pharmacology to screen out the active ingredients and targets of HLJDD. Secondly, we performed protein–protein interaction (PPI) networks both the HLJDD active ingredients related and OSCC related targets to get the core targets, and through biological process and pathway enrichment analysis of core targets to reveal the inhibitory mechanism of HLJDD on OSCC. Finally, we selected some core signal pathways and performed experimental verification in vitro.

## Methods

The whole workflow was illustrated in Graphical abstract. It shows the integrated workflow for elucidating the mechanism(s) of action of HLJDD in the treatment of OSCC. The workflow includes: 1. Data collection: the collection of potential components of HLJDD and their putative targets, the collection of OSCC-related targets; 2. Enrichment analysis: the identification of core targets and their enriched pathways; 3. Experimental validations of the important functions and pathways.

### Network pharmacology analysis

#### Databases

Traditional Chinese Medicine Systems Pharmacology Database and Analysis Platform (TCMSP) (http://lsp.nwu.edu.cn/tcmsp.php) [24]; Therapeutic Target Database (TTD) (http://bidd.nus.edu.sg/BIDD-Databases/TTD/TTD.asp) [25]; Uniprot (https://www.uniprot.org/) [26]; Online Mendelian Inheritance in Man (OMIM) (https://www.omim.org/) [27]; Search Tool for the Retrieval of Interacting Genes (STRING) (https://string-db.org/) [28, 29]; DAVID (https://david.ncifcrf.gov/summary.jsp) [30].

#### Software

Cytoscape v3.2.0 software (an open source software for visualizing complex biomolecular interaction networks, integrating high-throughput data and connecting the network to functions such as a feature annotation database [31]); Bisogenet plug-in of Cytoscape (a new tool for gene network building, visualization and analysis [32]); CytoNCA plug-in of Cytoscape (for the central analysis and evaluation of protein interaction networks [33]); ClueGo plug-in of Cytoscape (a convenient tool to do the enrichment analysis including Gene Ontology (GO), pathway annotation analysis, etc. [34]).

#### Prediction of target genes

Based on the TCMSP database, the active ingredients and related targets of *Coptis chinensis Franch*, *Scutellaria baicalensis Georgi*, *Phellodendron amurense Rupr* and *Gardenia jasminoides J.Ellis* in HLJDD were obtained and then exported. According to oral bioavailability more than 30%, we got potential active ingredients and targets of HLJDD. After screening, we transformed the protein names of targets to gene names by Uniprot Website (https://www.uniprot.org/). After preparing two files, we imported to Cytoscape v3.2.0 software and made HLJDD potential active ingredients-targets interaction network visualization. We searched OSCC related targets from Therapeutic Target Database (TTD) (http://bidd.nus.edu.sg/BIDD-Databases/TTD/TTD.asp) and Online Mendelian Inheritance in Man (OMIM) (https://www.omim.org/) database. After integrating these databases and deleting duplicates, we got final OSCC related targets. As showed above, protein names of OSCC related targets could be transformed to gene names (restricted to the Homo sapiens genus) by Uniprot Website (https://www.uniprot.org/) one by one.

#### Protein–protein interaction (PPI) network construction

HLJDD potential active ingredients related targets and OSCC related targets obtained in previous steps were imported into the Bisogenet plug-in of Cytoscape, so that five PPI network diagrams were obtained including *Coptis chinensis Franch*, *Scutellaria baicalensis Georgi*, *Phellodendron amurense Rupr*, *Gardenia jasminoides J.Ellis* potential active ingredients related targets and OSCC related targets, respectively. After merging these five PPI networks, we got an intersection network which called the HLJDD potential active ingredients related targets-OSCC related targets PPI network. Connecting with CytoNCA plug-in of Cytoscape, we evaluated the topological parameters and exported to a file. Then we selected the degree raw and calculated the median value. When imported twice the median value and the total number of nodes, the system automatically marked eligible nodes with yellow color which called core targets. The exported core targets could be used to build PPI network diagrams.

#### Enrichment analysis of core targets

ClueGO plug-in of Cytoscape was applied to analyze Gene Ontology (GO) and Kyoto Encyclopedia of Genes and Genomes (KEGG) for core targets, including Biological Process (BP), Cellular Component (CC) and Molecular Function (MF). Meanwhile, DAVID website (https://david.ncifcrf.gov/summary.jsp) was also used for GO and KEGG analysis in this study.

### Cell culture and experiment

#### Preparation of Huanglianjiedu Decoction (HLJDD) and control drugs

*Coptis chinensis Franch* (*Batch No*: 170901), *Scutellaria baicalensis Georgi* ((*Batch No*: 170801), and *Gardenia jasminoides J.Ellis* (*Batch No*: 170601) were provided by Anhui people’s Traditional Chinese Medicine Pieces Co., Ltd., *Phellodendron amurense Rupr* (*Batch No*: 170506) was provided by Hangzhou East China Traditional Chinese Medicine Pieces Co., Ltd. The Four TCM pieces were procured from ZhangTongtai Pharmacy. After the four pieces were powdered, we mixed *Coptis chinensis Franch*, *Scutellaria baicalensis Georgi*, *Phellodendron amurense Rupr* and *Gardenia jasminoides J.Ellis* in the ratio of 3:5:5:5, the formulation was decocted twice by refluxing with 1800 mL water for 60 min and 900 mL water for 30 min. Both the solutions were collected then concentrated by water-evaporation to 1 g/mL and stored at −20 °C [35]. Before administration, HLJDD solution was filtered with 0.22 μm filter and diluted by cell culture medium to 5 mg/mL, 2.5 mg/mL and 1.25 mg/mL HLJDD working concentrations respectively. Paclitaxel was purchased from Yangtze River Pharmaceutical (Jiangsu, China), the concentration of stock solution was 6 mg/mL. It was diluted to 6 μg/mL with cell culture medium before administration. Cisplatin was purchased from Qilu Pharmaceutical (Jinan, China) and stock solution (1 mg/mL) prepared with phosphate buffer saline (PBS). It was diluted to 5 μg/mL with cell culture medium before administration.

#### Cell culture

The CAL-27 human tongue squamous carcinoma cell (CRL-2095) and SCC-25 human oral squamous carcinoma cell line (CL-0569) were purchased from ATCC (Rockville, MD, USA). The CAL-27 cells were cultured in DMEM-High Glucose medium and SCC-25 cells were cultured in MEM medium, both supplemented with 100 U/mL penicillin, 100 μg/mL streptomycin and 10% fetal bovine serum (FBS) (Sigma-Aldrich, St. Louis, MO, USA) and incubated in 5% CO_2_ at 37 °C. Cells were seeded in 6-well plate or 96-well plate at the density of 4 × 10^5^ cells/mL. After 24 h, cells were treated with HLJDD (5 mg/mL, 2.5 mg/mL and 1.25 mg/mL), paclitaxel (6 μg/mL) and cisplatin (5 μg/mL), respectively. At the same time, without drug administration cells were used as the control group. After administration for 24, 48 and 72 h, cells in 96-well plate were harvested for Study Using sulforhodamine B (SRB) experiment. In addition, cells in 6-well plate were harvested after administration for 24 h to do wound healing assay, western blot analysis, flow cytometry of cell cycle and apoptosis.

#### Wound healing assay

Cells were seeded into 6-well plates at the density of 4 × 10^5^ cells/mL and cultured for 24 h at 37 °C. After reaching 90–100% confluence, the cells were scraped vertically using sterile 1000 µL-pipette tips and washed with PBS to remove cellular debris. The cells were treated with HLJDD (5 mg/mL, 2.5 mg/mL and 1.25 mg/mL), paclitaxel (6 μg/mL) and cisplatin (5 μg/mL) respectively and then incubated in fresh serum-free medium for 24 h. Images were collected at 0 and 24 h under an inverted microscope (Olympus, Japan). The area measurement was analyzed quantitatively by Image J software (National Institute of Health, USA) [36].

#### Western Blot Analysis

Cells were seeded into 6-well plates at the density of 4 × 10^5^ cells/mL and cultured for 24 h at 37 °C. Then cells were treated with HLJDD (5 mg/mL, 2.5 mg/mL and 1.25 mg/mL), paclitaxel (6 μg/mL) and cisplatin (5 μg/mL) respectively. After 24 h incubation, cells in 6-well plate were harvested and the proteins of cells were extracted with RIPA buffer (containing protease inhibitor cocktail, phosphatase inhibitor and phenylmethanesulfonyl fluoride (PMSF) and centrifuged at 12,000×*g* at 4 °C for 15 min. Protein concentrations were detected by Bio-Rad reagent (Bio-Rad Inc., Hercules, CA, USA). The 5 × loading buffer (Beyotime, Shanghai, China) was added to proteins and boiled at 100 °C for 5 min. 30 μg of proteins were separated by electrophoresis on SDS-PAGE gels (30 V for 30 min, 70 V for 40 min and 130 V for 30 min), then gels were transferred to the nitrocellulose (NC) membrane (300 mA for 90 min). After transferring time, the membranes were blocked for 60 min with 5% bovine serum albumin (BSA) then incubated with the primary antibody (p-ERK1/2 (ab76165, 1:3000, Abcam, U.K.), ERK1/2 (ab79853, 1:1000, Abcam, U.K.), p-NF-κB p65 (S468) (AF3390, 1:1000, CST, USA), NF-κB p65 (BS1253, 1:1000,Bioworld, USA), p-IKK(α + β) (S180 + S181) (ab55341, 1:1000, Abcam, U.K.), GAPDH (db106, 1:50000, Diagbio, China), p-AKT (#4060, 1:1000, CST, USA), AKT1/2/3 (db1607, Diagbio, China), p-NF-κB p65 (S529) (BS4737, 1:1000, Bioworld, USA), p-NF-κB p65 (S276) (BS4135, 1:1000, Bioworld, USA), p-p38 (#4511, 1:1000, CST, USA), p38 (db3666, 1:1000, Diagbio, China), p-JNK (#9255, 1:1000, CST, USA) and JNK (#9252, 1:1000, CST, USA)) at 4 °C overnight. After that, membranes were washed six times for 4 min each time with TBST and incubated with the secondary antibody (IRDye 800CW anti-rabbit (926–32211, 1:5000, LI-COR Biosciences, USA) and IRDye 680RD anti-mouse (926–68070, 1:5000, LI-COR Biosciences, USA) at room temperature for 90 min. Lastly membranes were washed six times for 4 min each time with TBST and imaged with Odyssey CLx infrared imaging system (LI-COR Biosciences, USA). The bands were quantified by Imagine Studio Version 5.2 software (LI-COR Biosciences, USA) and total proteins or GAPDH were used to normalize the target proteins.

#### Cell proliferation determination by sulforhodamine B (SRB)

Cell proliferation was measured by SRB kit (KeyGEN BioTECH, Nanjing, China). Cells were seeded in 96-well plate at the density of 1 × 10^3^ cells/ well for 24 h. Then the cells were treated with HLJDD (5 mg/mL, 2.5 mg/mL and 1.25 mg/mL), paclitaxel (6 μg/mL) and cisplatin (5 μg/mL) respectively for the next 24, 48 and 72 h. After incubation, the cells were washed with 1 × PBS and fixed with 100 µL 50% TCA for 1 h at 4 °C. Then every 30 s, we used 100 µL washing solution A to wash cells for 2 times. 50 µL of SRB dying solution was added to each well and incubated for 15 min at room temperature, protected from light. We used 100 µL washing solution B to remove the unbound SRB and repeated it 5–10 times. 100 µL dissolving solution was added and incubated at room temperature for 5 min, protected from light. Absorbance was measured at 515 nm. The inhibitory rate of cell proliferation was calculated according to the formula: ((control OD value-experimental OD value)/control OD value) × 100% [37].

#### Cell cycle detection by flow cytometry analysis

Cells were harvested after administration for 24 h and washed with cold PBS for two times. After fixed with ice-cold 70% ethanol at 4 °C overnight, cells were centrifuged at 2000 rpm for 5 min and washed with cold PBS. Then cells were added with 500 μL PBS which containing 50 μg/mL Ethidium Bromide (EB), 100 μg/mL RNase A and 0.2% Triton X-100. After incubation at 4 °C for 30 min in the dark place, DNA contents were evaluated by CytoFLEX LX flow cytometry analyzer (Beckman Coulter, Inc. 250S. Kraemer Boulevard Brea, CA 92,821, USA). The result was analyzed by Denovo FCS Express 6 software (De Novo Software, 207 N. Sierra Madre Blvd, Pasadena, CA 91,107, USA).

#### Cell apoptosis measurement by flow cytometry analysis

Cells were seeded in 6-well plates and treated with HLJDD (5 mg/mL, 2.5 mg/mL and 1.25 mg/mL), paclitaxel (6 μg/mL) and cisplatin (5 μg/mL) respectively for 24 h. Cell apoptosis was measured by a FITC Annexin V Apoptosis Detection Kit (556547, BD Biosciences, San Jose, California, USA). Cells were washed twice with 1 × cold PBS and stained with 5 µL Annexin V-FITC and 5 µL PI. After incubation at 4 °C in the dark place for 20 min. The apoptosis was analyzed by DxFLEX flow cytometry analyzer (Beckman Coulter, Inc. 250S. Kraemer Boulevard Brea, CA 92821, USA).

### Statistical analysis

All data were expressed as the mean ± standard error of mean (SEM). Statistical differences among the different groups were evaluated by one-way ANOVA with Bonferroni’s multiple comparison test with Graph Prism 5 software (GraphPad Software Inc., San Diego, CA, USA). *p* < 0.05 was considered significant.

## Results

### Construction of HLJDD potential active ingredients-targets interaction network

**Fig. 1:**
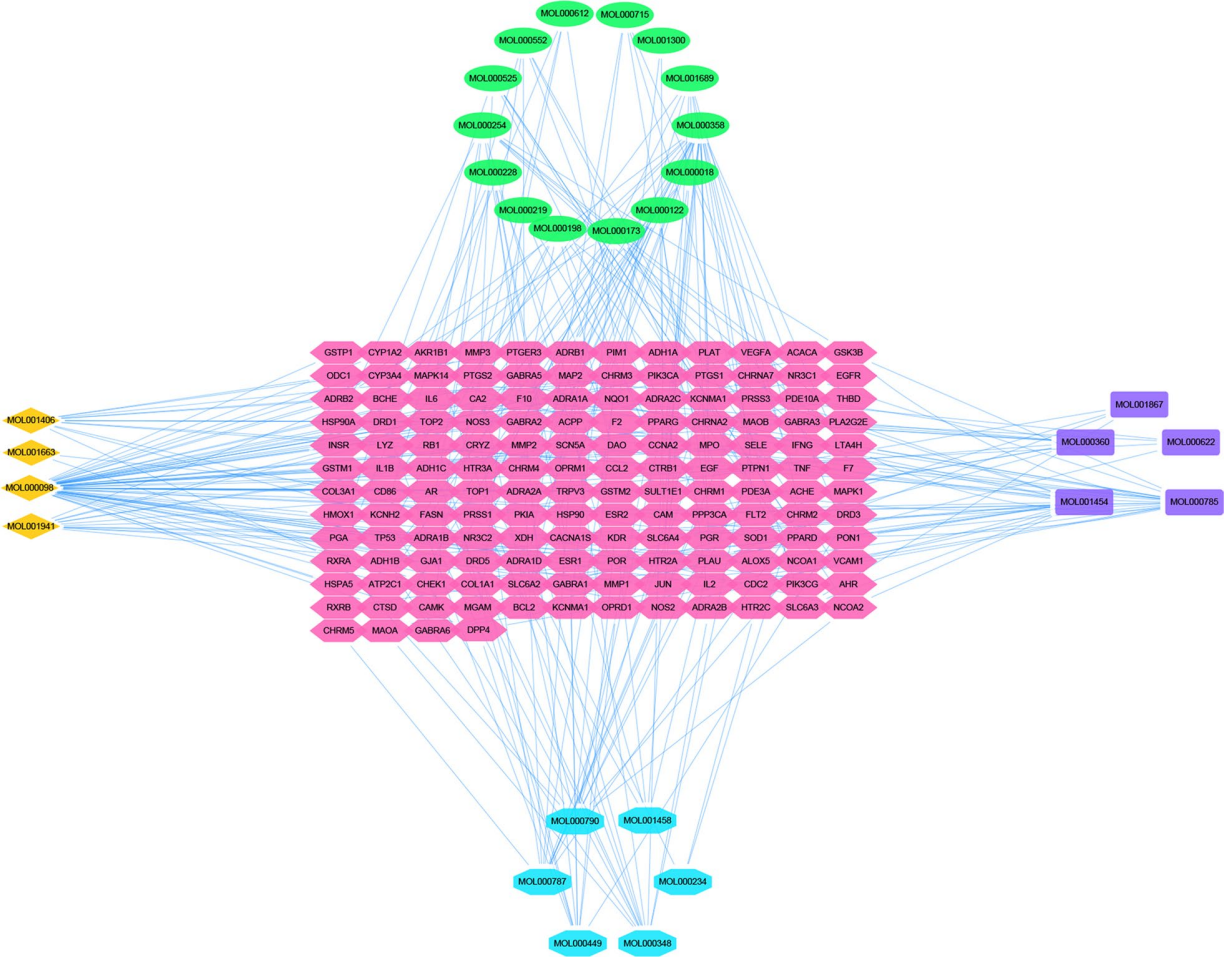
Potential active ingredients-targets interaction network of HLJDD. We built a data file and a property file, and then imported into Cytoscape software to generate a HLJDD potential active ingredient-target network diagram. Green color represented the potential active ingredient of *Scutellaria baicalensis Georgi*; purple color represented the potential active ingredient of *Coptis chinensis Franch*; yellow color represents the potential active ingredient of *Gardenia jasminoides J.Ellis*; blue color represented the potential active ingredient of *Phellodendron amurense Rupr* and the middle pink color represented the common targets of potential active ingredients related targets

The effect of TCM formula on disease depends on the relationship between the effective ingredients and their targets. We found out 35 HLJDD potential active ingredients (Table 1), 5 in *Coptis chinensis Franch*, 16 in *Scutellaria baicalensis Georgi*, 10 in *Phellodendron amurense Rupr* and 4 in *Gardenia jasminoides J.Ellis*. In the Table 1, the repeated potential active ingredients between *Coptis chinensis Franch* and *Phellodendron amurense Rupr* were MOL000622, MOL000785 and MOL001454. Moreover, the repeated potential active ingredients between *Scutellaria baicalensis Georgi* and *Phellodendron amurense Rupr* was MOL000358. Previous data showed that ingredients such as Coptisine, Palmatine, berberine, wogonin, etc. had been confirmed in mass spectrometry (MS) and MS/MS data experiments to identify the prototype components in rat plasma after oral administration of HLJDD [38] and latter three ingredients were well separated by High Performance Liquid Chromatography (HPLC) fingerprint method [39]. It indicated that the potential active ingredients we had obtained were worthy of further research. Then we searched related targets of HLJDD potential active ingredients. The number of related targets of *Coptis chinensis Franch*, *Scutellaria baicalensis Georgi*, *Phellodendron amurense Rupr* and *Gardenia jasminoides J.Ellis* were 83, 348, 284 and 117, respectively. Because there were many duplicate targets in the ingredients of TCM, we had recounted the number of targets related to the potential active ingredients of *Coptis chinensis Franch*, *Scutellaria baicalensis Georgi*, *Phellodendron amurense Rupr* and *Gardenia jasminoides J.Ellis* which were 39, 93, 81 and 88, respectively. To visualize the relationship, we built the potential active ingredients-related targets interaction network by Cytoscape software. As it showed in Fig. 1, different color represented potential active ingredients of *Coptis chinensis Franch*, *Scutellaria baicalensis Georgi*, *Phellodendron amurense Rupr* and *Gardenia jasminoides J.Ellis*. The middle pink color part represented the common targets set of potential active ingredients related targets. For the duplicated targets corresponding to different potential active ingredients in HLJDD, this figure only showed the one target which linked related potential active ingredients.

**Table 1:**
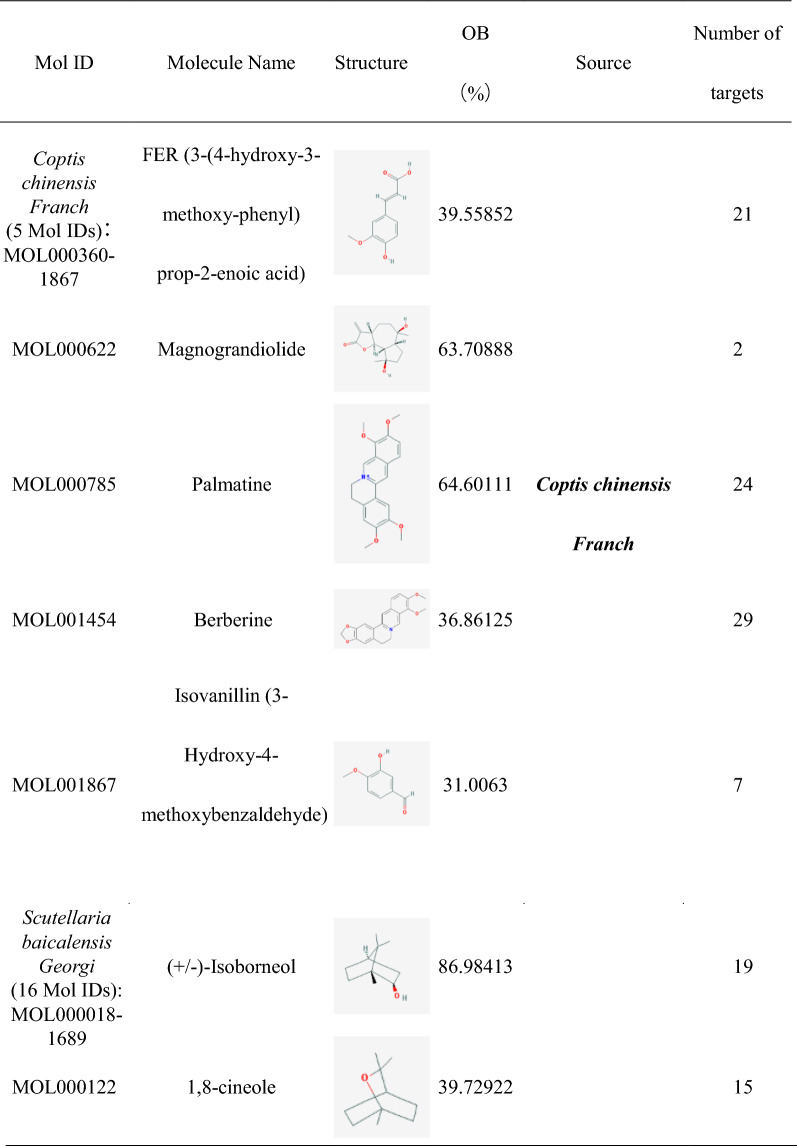

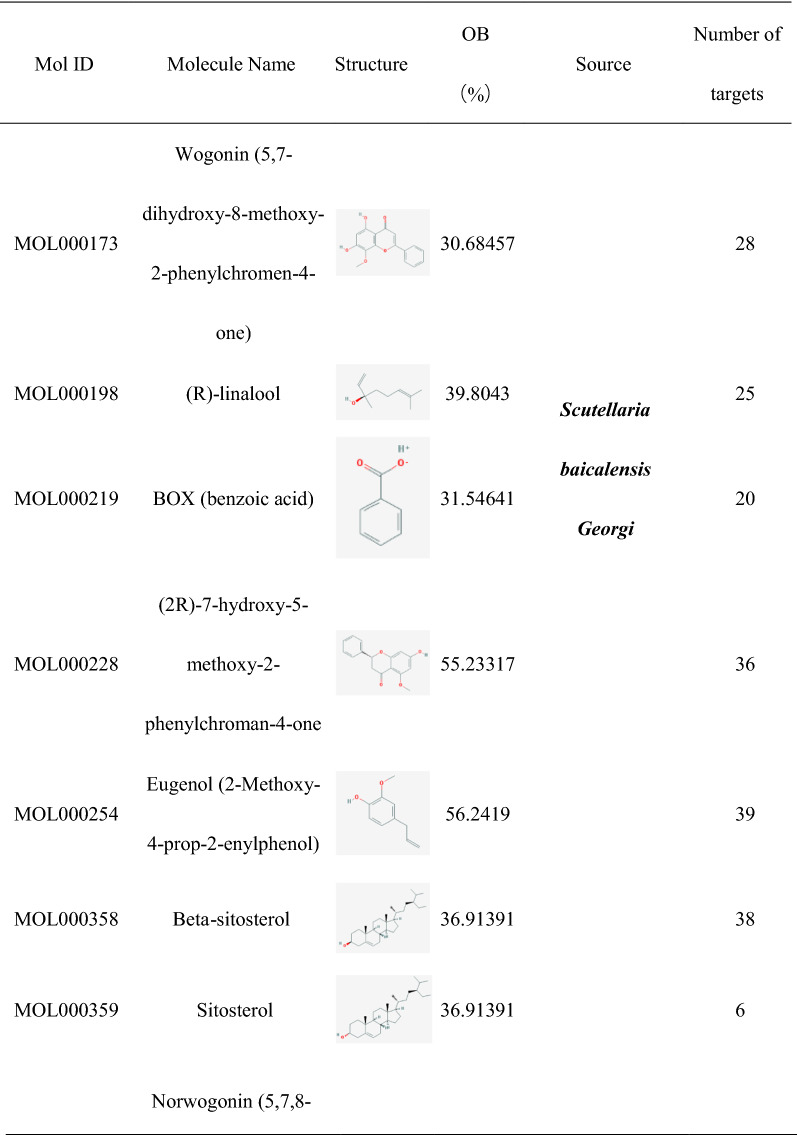

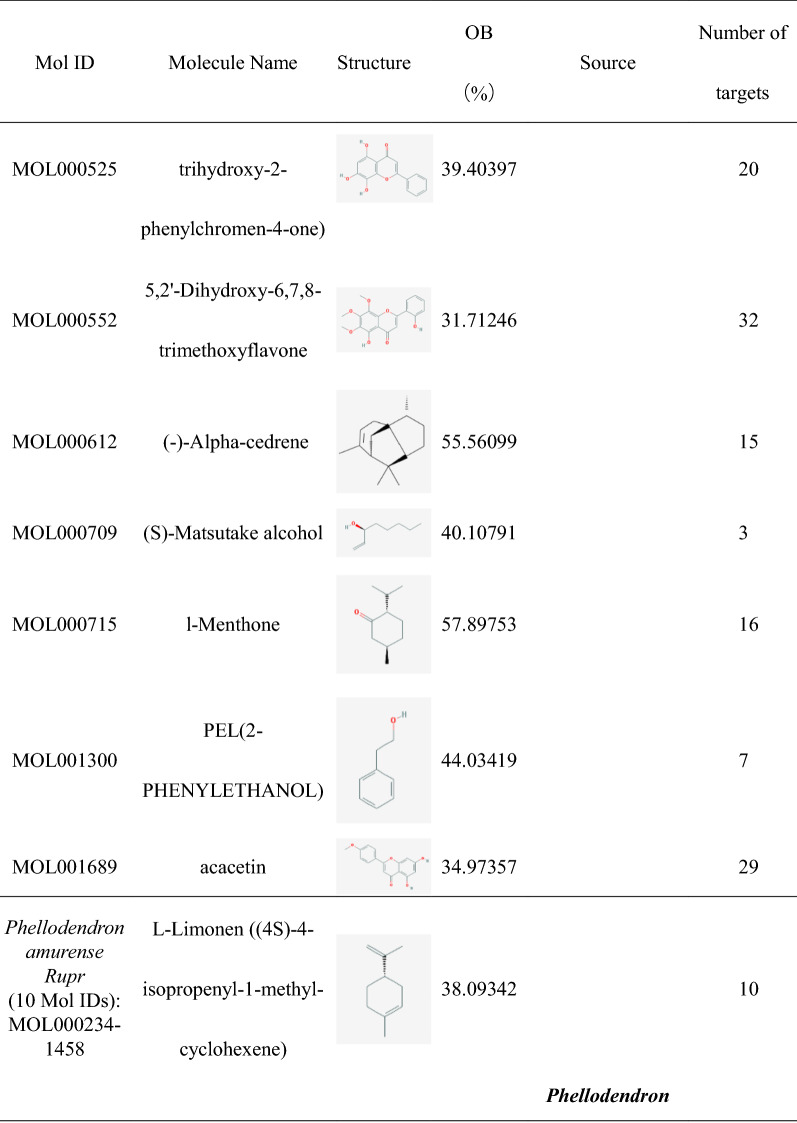

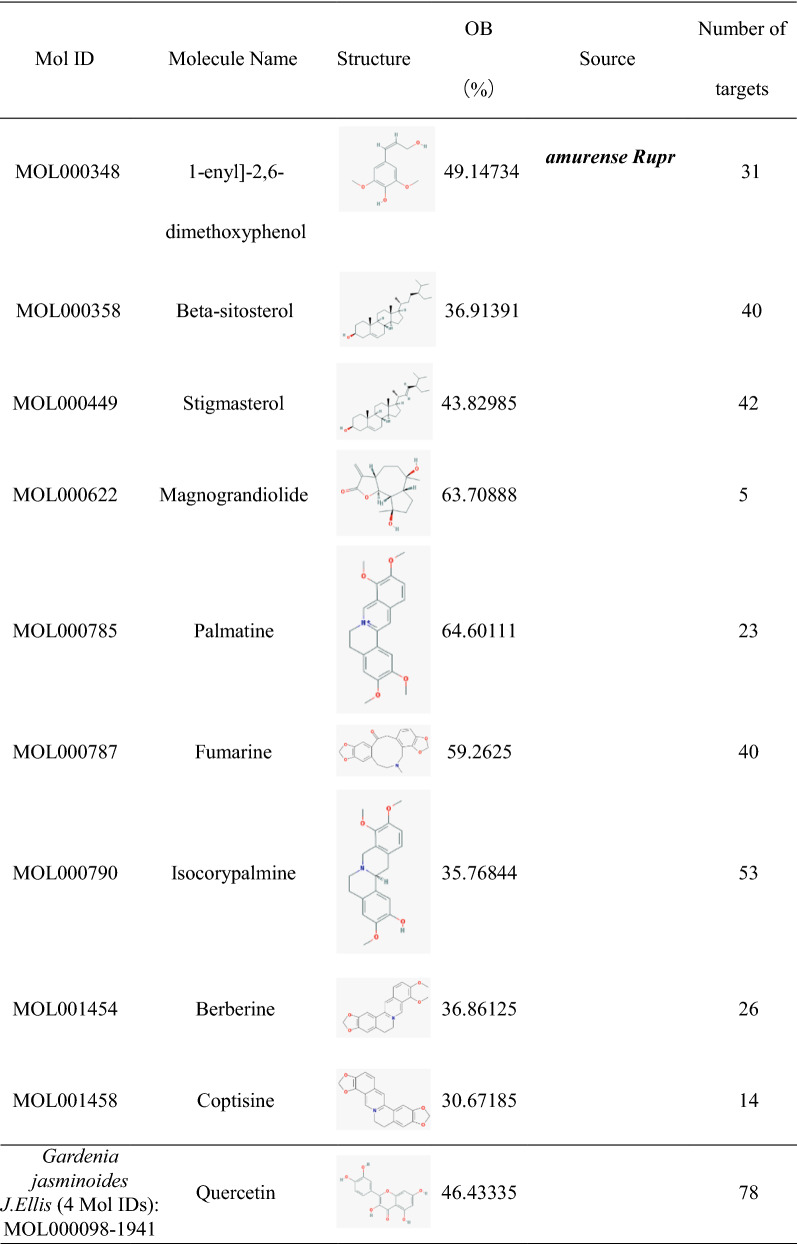

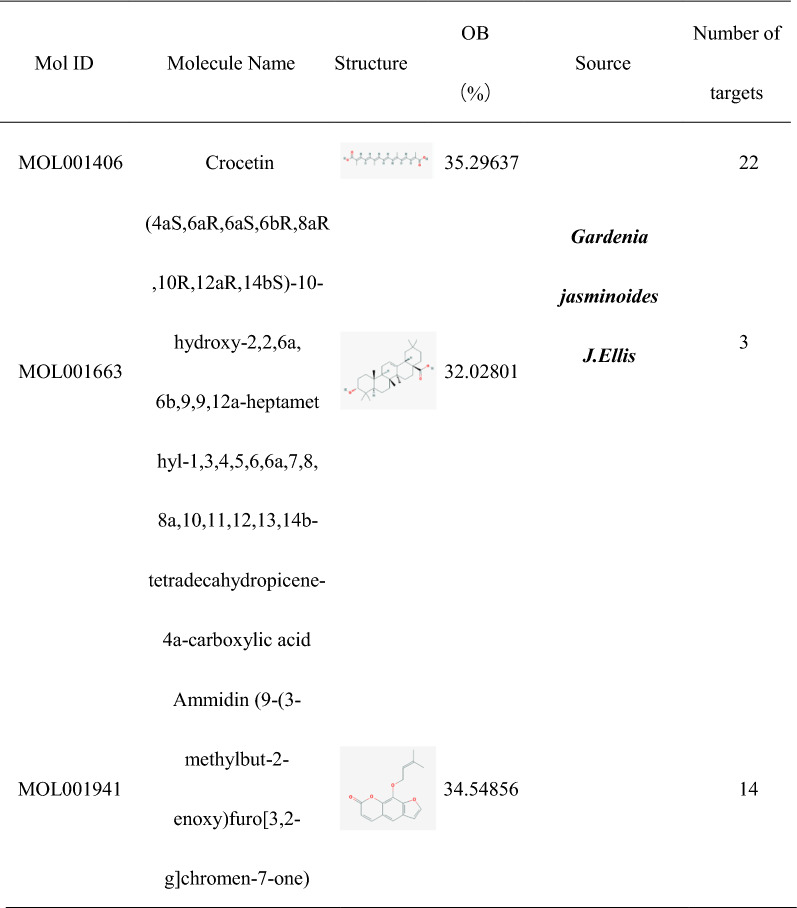
Potential active ingredients and the number of their related targets in HLJDD formula

### Screening the core targets between HLJDD potential active ingredients related targets and OSCC related targets

**Fig. 2:**
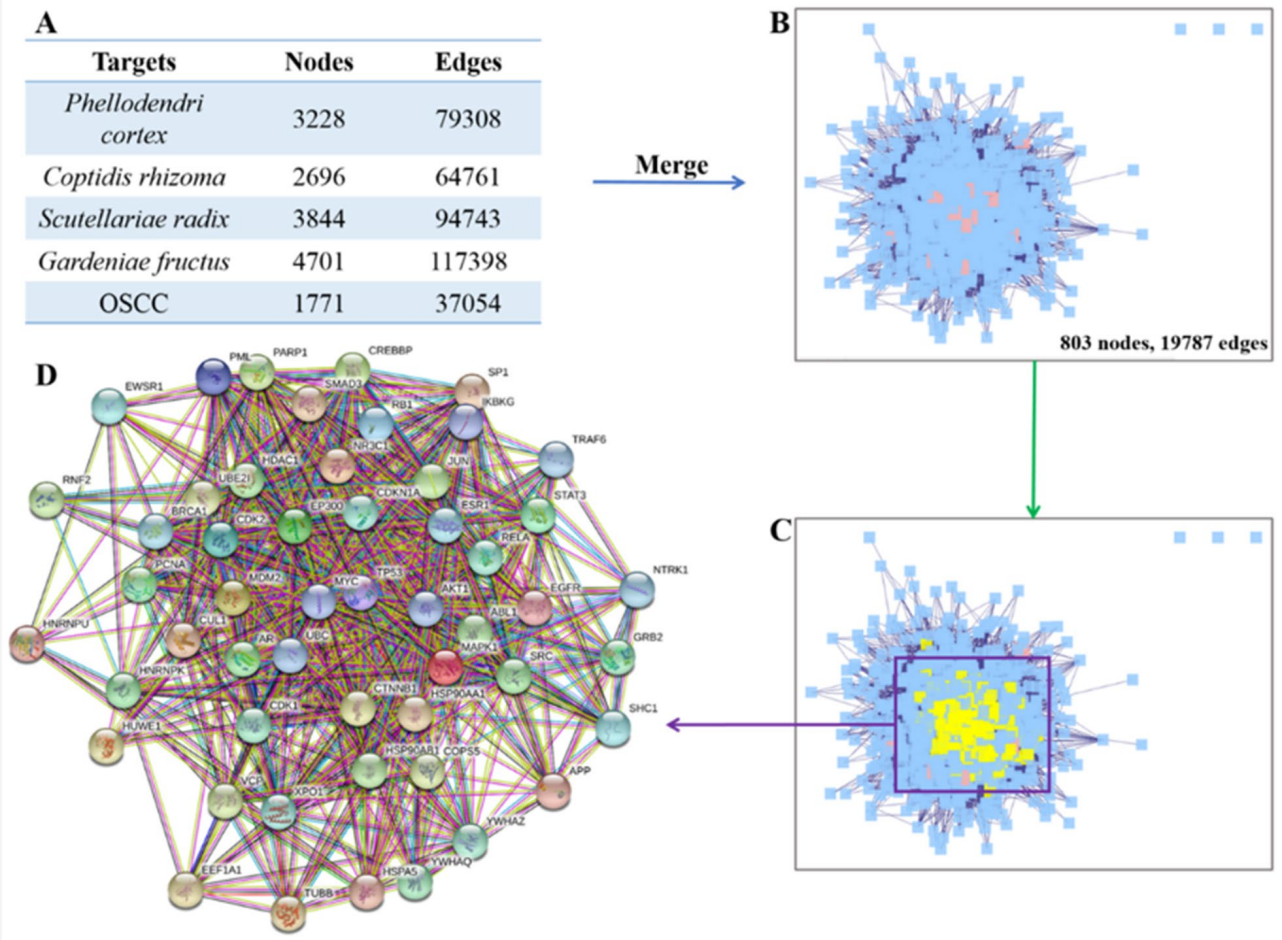
Identification of core targets for HLJDD against OSCC. **A** The nodes and edges of five networks including OSCC related targets, the potential active ingredients related targets of *Phellodendron amurense Rupr, Coptis chinensis Franch*, *Scutellaria baicalensis Georgi* and *Gardenia jasminoides J.Ellis.* **B** A merged network. **C** The core targets screening by degree value. Yellow color represented the core targets. **D** The protein–protein interaction network of the core targets built by using Search Tool for the Retrieval of Interacting Genes (STRING) (https://string-db.org/). Network nodes represented proteins and edges represented protein–protein associations (Different colors represented that protein–protein interactions validated by different sources)

We used Bisogenet in Cytoscape to make PPI network diagrams including OSCC related targets, HLJDD potential active ingredient related targets of *Phellodendron amurense Rupr, Coptis chinensis Franch*, *Scutellaria baicalensis Georgi* and *Gardenia jasminoides J.Ellis.* In Fig. 2A, the nodes of *Phellodendron amurense Rupr, Coptis chinensis Franch*, *Scutellaria baicalensis Georgi*, *Gardenia jasminoides J.Ellis* and OSCC related targets were 3228, 2696, 3844, 4701 and 1771, respectively. The number of edges were 79,308, 64,761, 94,743, 117,398 and 37,054, respectively. Through the Merge Function in the toolbar of Cytoscape, we got the intersection network diagram of these five PPIs which contained 803 nodes and 19,787 edges.

After performing a topological analysis of the network in the CytoNCA plug-in of Cytoscape, we found that each gene had almost 48.174 neighbors (Table 2). After calculating median of degree value, we imported twice the median value and the total number of nodes, the system automatically marked eligible nodes with yellow color which called core targets (Fig. 2B and C). Finally, we obtained 52 core targets which were more than twice the median of degree value (Table 3). The greater of degree value meant the more nodes interaction with this gene. Moreover, we also rebuilt the core target PPI network diagram (Fig. 2D).

**Table 2:** The topological parameters of merged network (HLJDD potential active ingredients related targets—OSCC related targets protein–protein interaction network)

Parameters	Results	Parameters	Results
Clustering coefficient	0.284	Number of nodes	803
Connected components	4	Network density	0.060
Network diameter	4	Network heterogeneity	0.910
Network radius	3	Isolated nodes	3
Network centralization	0.581	Number of self-loops	445
Shortest paths	639200 (99%)	Multi-edge node pairs	0
Characteristic path length	2.070	Analysis time (sec)	0.446
Avg. number of neighbors	48.174

**Table 3:** The core targets of merged network (HLJDD potential active ingredients related targets-OSCC related targets protein–protein interaction network)

Name	Degree value	Name	Degree value	Name	Degree value
RNF2	61	SHC1	59	RELA	75
GRB2	87	ABL1	66	HSP90AB1	86
NR3C1	71	CΜL1	73	PARP1	64
MDM2	93	STAT3	65	JUN	77
CREBBP	73	BRCA1	93	PML	59
COPS5	75	HUWE1	72	AKT1	72
SRC	75	MAPK1	60	CTNNB1	78
UBC	93	NTRK1	152	CDK1	56
UBE2I	70	EEF1A1	55	TP53	225
PCNA	55	EGFR	126	CDK2	102
EP300	97	IKBKG	61	EWSR1	64
AR	195	XPO1	64	CDKN1A	64
APP	80	YWHAZ	79	TRAF6	66
SP1	59	HNRNPK	54	SMAD3	70
YWHAQ	54	HNRNPU	54	ESR1	144
HDAC1	73	HSPA5	67	MYC	94
VCP	79	HSP90AA1	101		
TUBB	67	RB1	67		

### Enrichment analysis of core targets

**Fig. 3:**
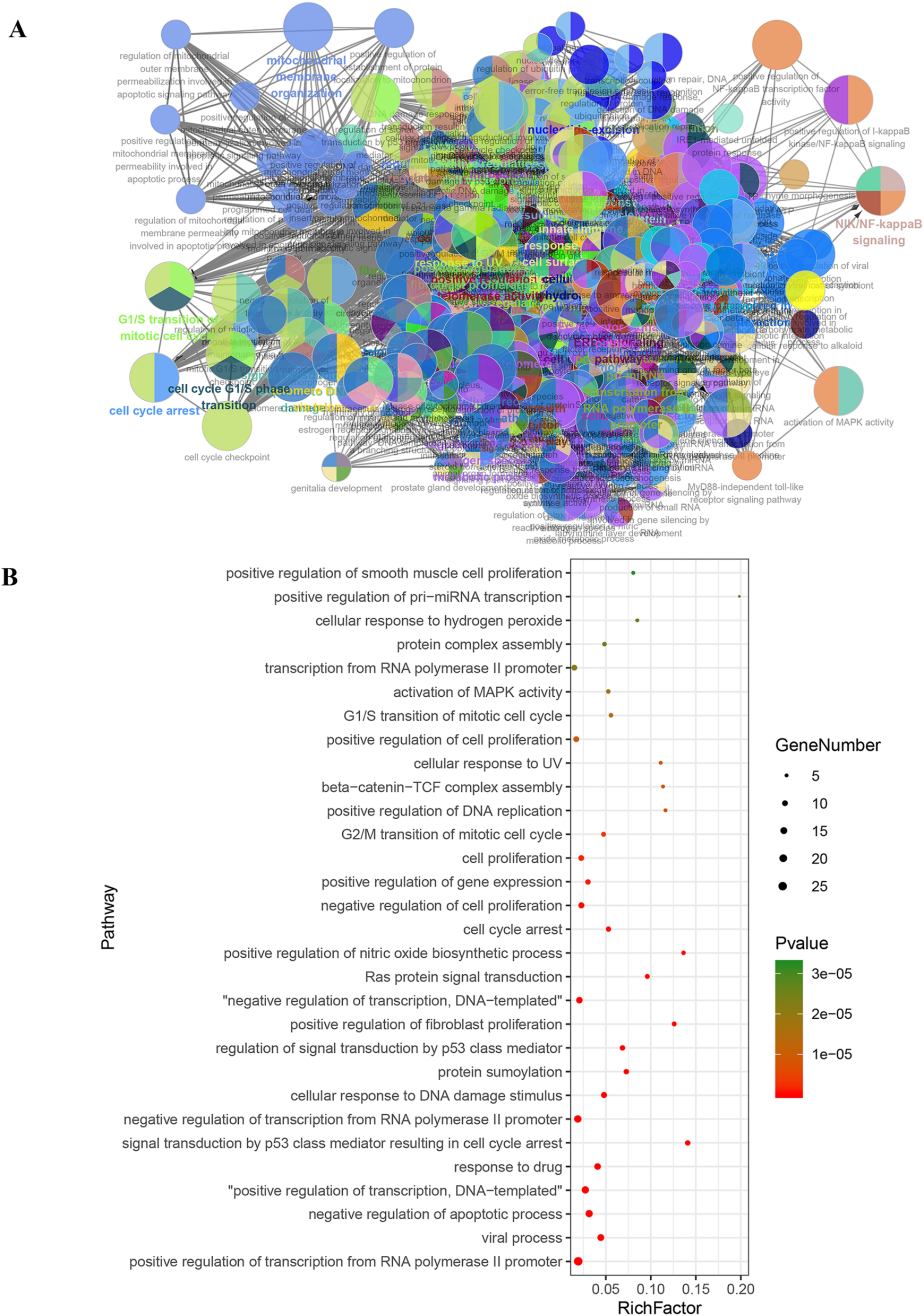

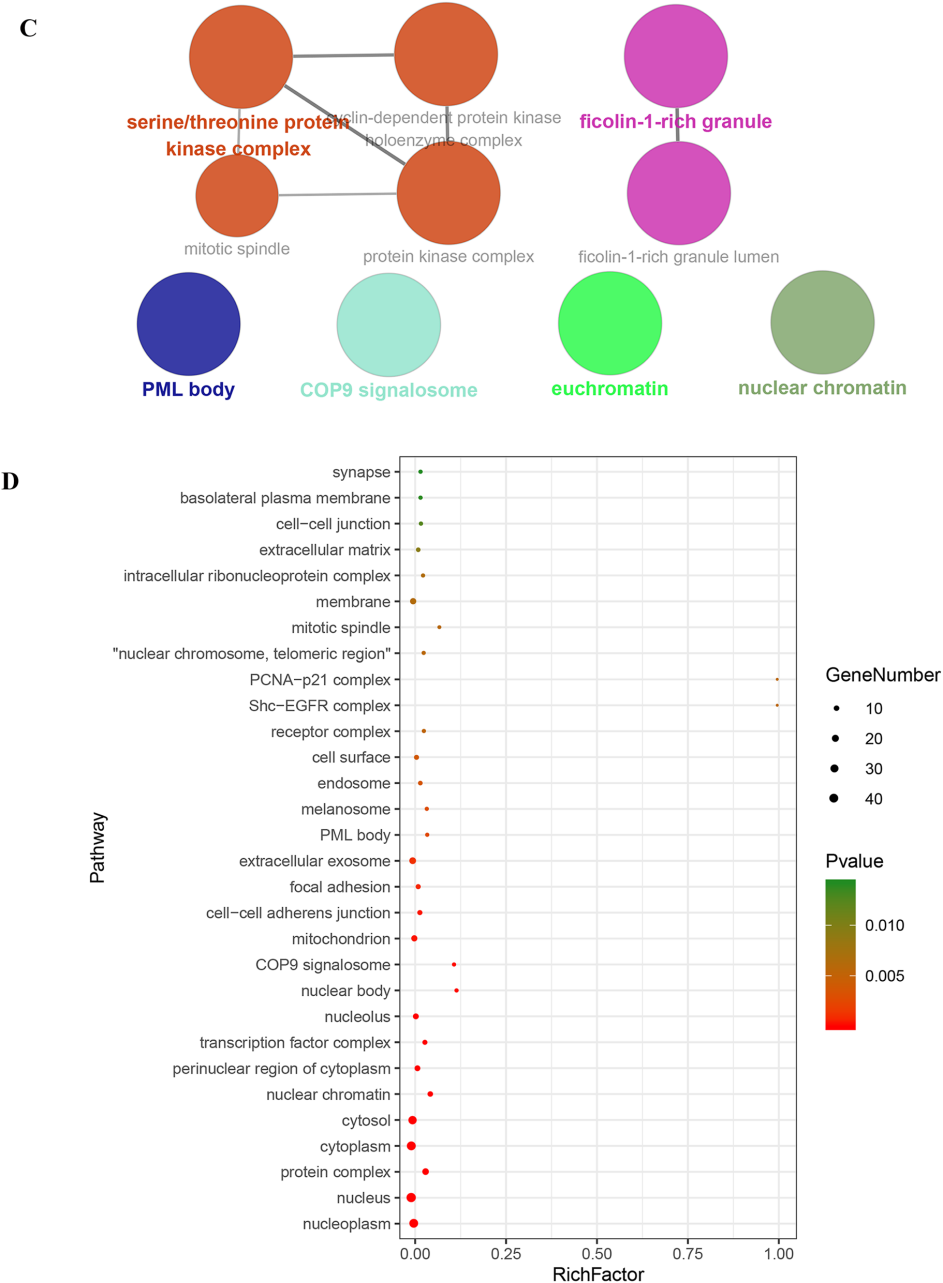

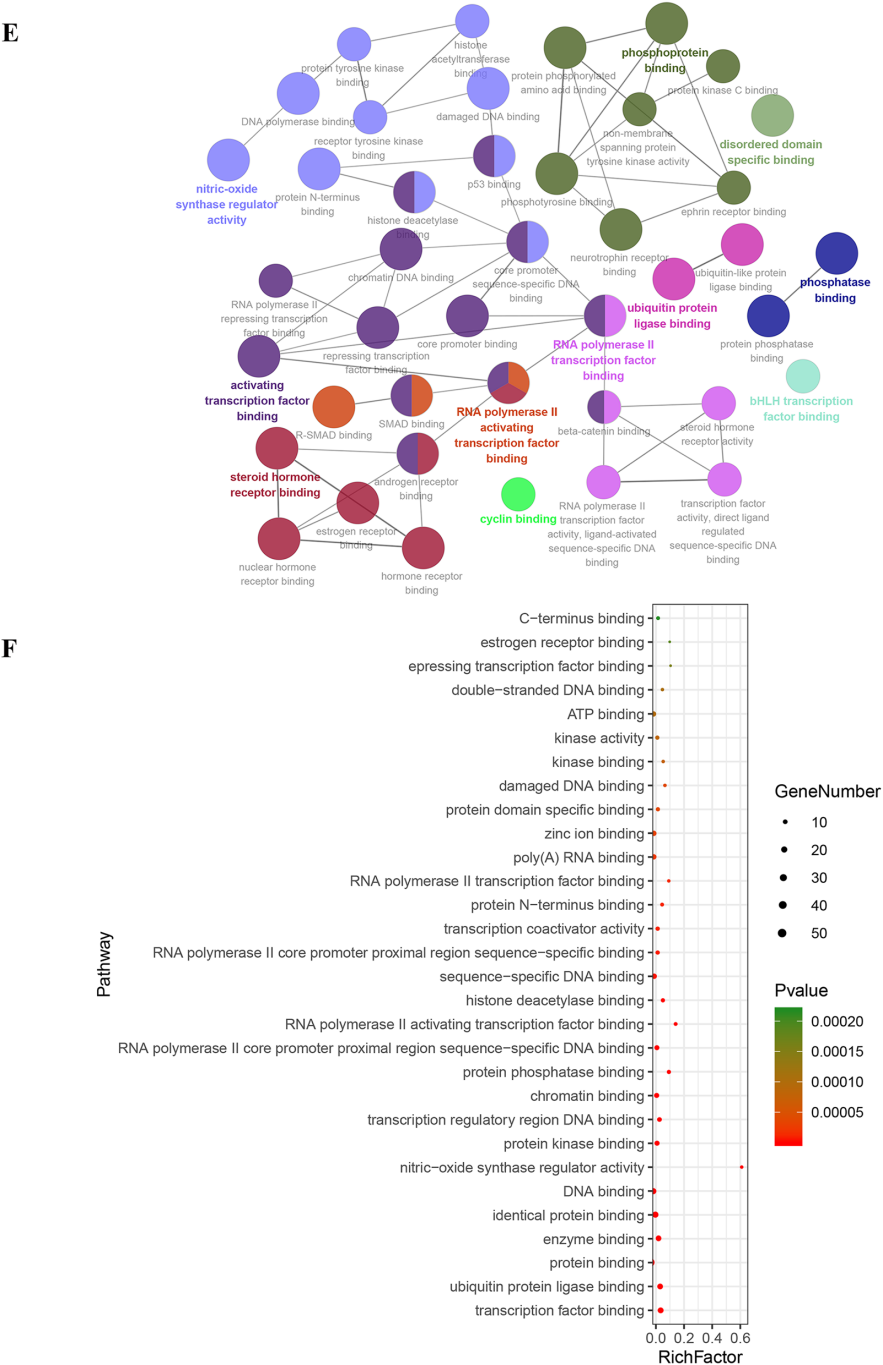

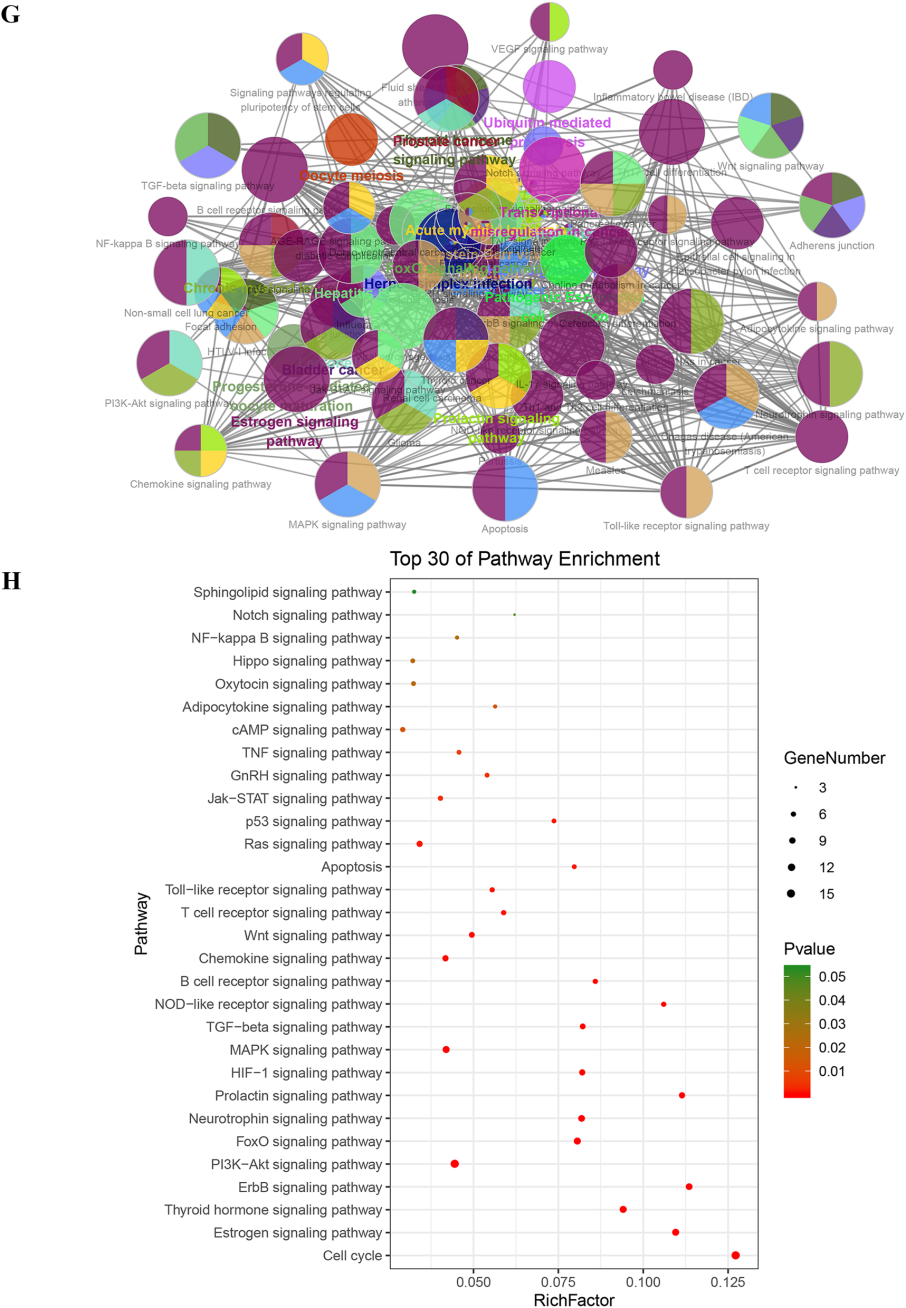
The enrichment analysis of Gene Ontology (GO) and Kyoto Encyclopedia of Genes and Genomes (KEGG) pathway. **A** Biological process results in GO enrichment analysis (from ClueGO). Different colors represented different biological processes. If a gene was involved in different biological processes, it would show the proportion of different colors in the pie chart. **B** Biological process results in GO enrichment analysis (from DAVID). Rich Factor represented the degree of enrichment. The size of the bubble represented the number of genes enriched in one kind of biological process and the color represented the *p* value. The smaller *p* value represented enrichment more significant. **C** Cellular component results in GO enrichment analysis (from ClueGO). **D** Cellular component results in GO enrichment analysis (from DAVID). **E** Molecular function results in GO enrichment analysis (from ClueGO). **F** Molecular function results in GO enrichment analysis (from DAVID). **G** KEGG enrichment analysis results (from ClueGO). **H** Selected pathway results of KEGG enrichment analysis (from DAVID)

Using above exported core targets, we combined ClueGO plug-in in Cytoscape and DAVID website (https://david.ncifcrf.gov/summary.jsp) for enrichment analysis including Gene Ontology (GO) (Biological Process (BP), Cellular Component (CC) and Molecular Function (MF)) and Kyoto Encyclopedia of Genes and Genomes (KEGG) analysis (Fig. 3G). GO analysis of core targets was performed at *p*V ≤ 0.05, containing GO-BP (Fig. 3A), GO-CC (Fig. 3C) and GO-MF (Fig. 3E). Because we defined the filter condition of pV ≤ 0.05 before doing the enrichment analysis, the specific *p* value was not displayed in the exported data. But the DAVID website just made up for this shortcoming. After exporting *p* values from DAVID website, we sorted them from the smallest to the largest and took the TOP 30 to make advanced bubble charts of GO-BP (Fig. 3B), GO-CC (Fig. 3D), GO-MF (Fig. 3F) and KEGG pathway analysis (Fig. 3H).

In Fig. 3A and B, we got 1156 items from ClueGO and 296 items from DAVID website for GO-BP. Based on top 30 biological processes presented in Fig. 3B, it suggested that HLJDD might involve in some typical biological processes, such as negative regulation of apoptotic processes; cell cycle arrest; G2 / M switch of cycle; positive regulation of DNA replication; G1/S switch of mitotic cell cycle; activation of MAPK activity, etc. As for GO-CC, there were 10 items from ClueGO and 48 items from DAVID website (Fig. 3C and D). It revealed that HLJDD might take part in some CC, such as mitotic spindle, nuclear chromosome, telomeric region, etc. Moreover, we got 51 items from ClueGO and 101 items from DAVID website for GO-MF. According to the TOP30, HLJDD might affect transcription factor binding, protein kinase binding, kinase binding and activity, etc. (Fig. 3E and F). Based on the enrichment of these molecular function, it was necessary to detect of protein phosphorylation and kinase activity in the further experiments. KEGG pathway enrichment results indicated that HLJDD might work through cell apoptosis pathway, PI3K-AKT, mitogen-activated protein kinase (MAPK) and nuclear factor-kappa B (NF-κB) etc. signaling pathway to produce the anti-tumor effect on OSCC. Taken together, enrichment analysis of core targets provided the experimental directions for future verification.

### The inhibitory effect of HLJDD on the growth of OSCC cell lines

**Fig. 4:**
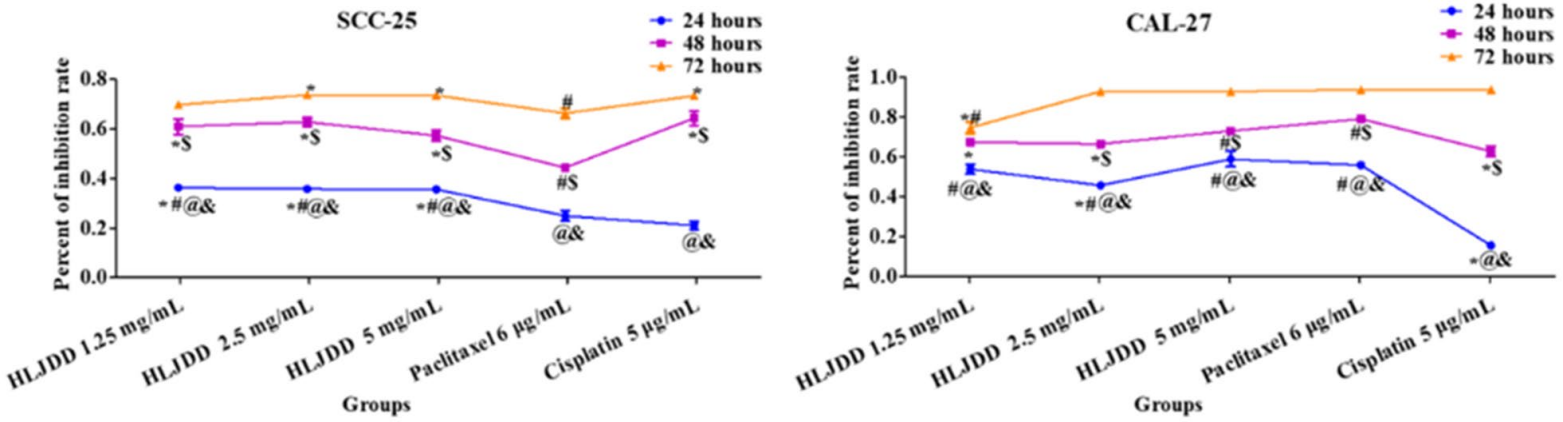
The inhibitory effects of HLJDD, paclitaxel and cisplatin on SCC-25 and CAL-27 cell lines were detected by sulforhodamine B (SRB) measurement after administration for 24, 48 and 72 h. n = 6. * *p* < 0.05 vs. paclitaxel group in different time points. # *p* < 0.05 vs. cisplatin group in different time points. @ *p* < 0.05 24 h vs. 48 h. $ *p* < 0.05 48 h vs. 72 h. & *p* < 0.05 24 h vs. 72 h

With the measurement of sulforhodamine B (SRB), we detected the inhibitory rate of HLJDD on CAL-27 and SCC-25 OSCC cell lines. In Fig. 4, the inhibitory effects of HLJDD (1.25 mg/mL, 2.5 mg/mL, 5 mg/mL), 6 μg/ mL paclitaxel and 5 μg/ mL cisplatin on cells were increased in time dependent manners. But there was no dose-dependent change on HLJDD. Compared with paclitaxel or cisplatin, HLJDD had similar inhibitory effect and after administration 24 h the effects of three concentrations of HLJDD on SCC cells were higher than paclitaxel or cisplatin. Therefore, it was worth to detect the further mechanism after administration for 24 h with three different concentrations of HLJDD and two positive drugs (paclitaxel and cisplatin) on SCC-25 and CAL-27 cell lines.

### HLJDD promoted late stage of cell apoptosis

**Fig. 5:**
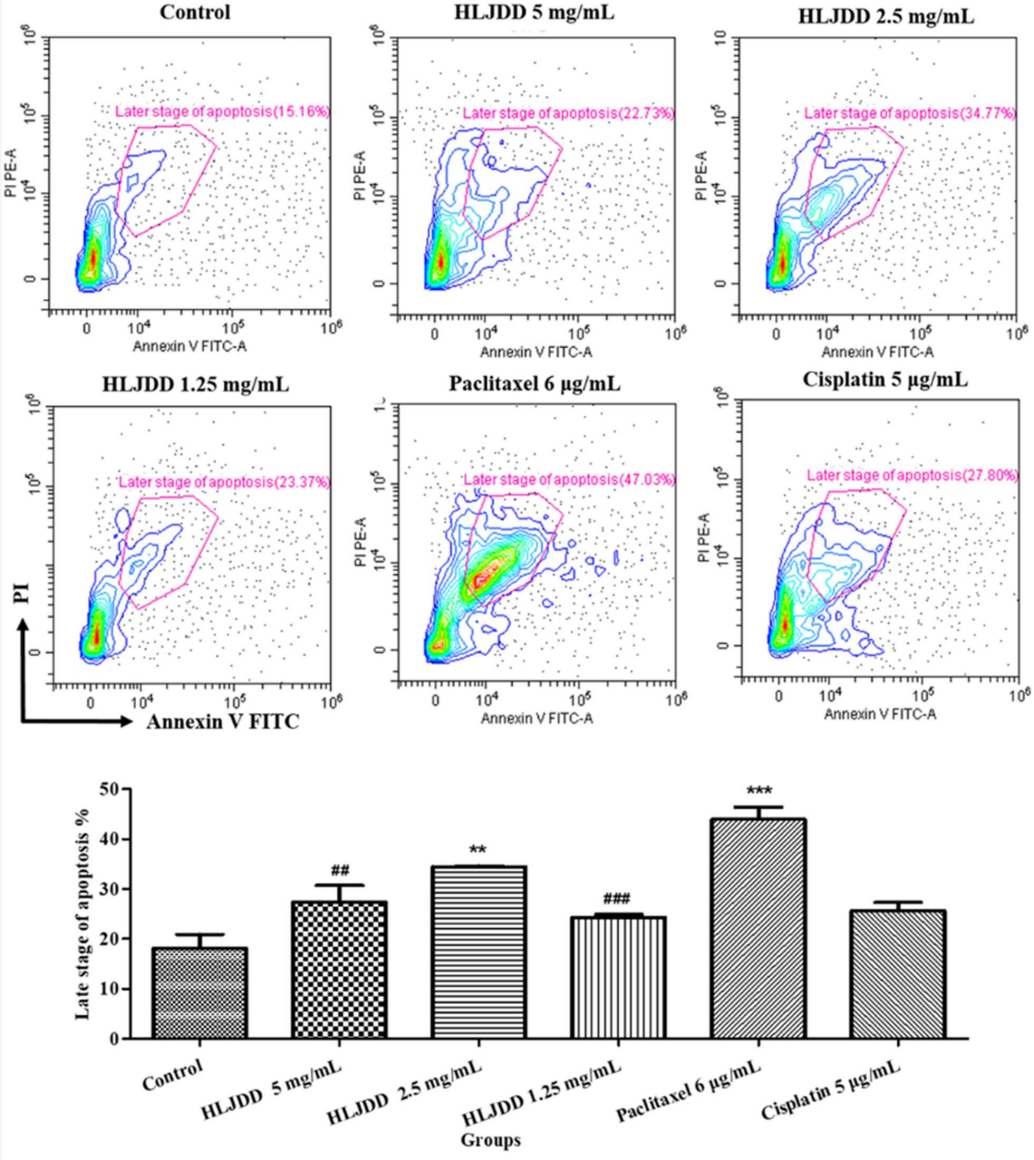
The representative contour plots of six groups and the quantitative statistical chart. SCC-25 cells were treated with HLJDD 5 mg/mL, HLJDD 2.5 mg/mL, HLJDD 1.25 mg/mL, paclitaxel 6 μg/mL and cisplatin 5 μg/mL for 24 h. Late stage of apoptosis determined by DxFLEX flow cytometry analyzer following Annexin V FITC and Propidium Iodide (PI) staining. Both Annexin V FITC and PI positive cells represented that the cells were in late stage of apoptosis. The statistics were expressed as Mean ± SEM, n = 3. *** *p* < 0.001, ** *p* < 0.01 vs. control group. ### *p* < 0.001, ## *p* < 0.01 vs. paclitaxel group. There were no significant differences between other groups and cisplatin group

The result of SRB indicated that HLJDD inhibited proliferation of OSCC cell lines. Based on the enrichment results, we proposed HLJDD may involve in cell cycle and apoptosis, then inhibit the proliferation of OSCC cell lines. We performed the Annexin-V FITC/PI staining to detect the apoptosis of SCC-25 cells after administration for 24 h (Fig. 5). Comparing with control group, percentages of late-stage apoptosis in 2.5 mg/mL HLJDD group and paclitaxel group were significantly increased, while others were increased without significance. Interestingly, the percentage of late-stage apoptosis in 2.5 mg/mL HLJDD group was higher than that in 1.25 mg/mL and 5 mg/mL HLJDD groups, and even cisplatin group. Compared to paclitaxel group, the percentages of late-stage apoptosis in 1.25 mg/mL and 5 mg/mL HLJDD groups were significantly reduced, while in 2.5 mg/mL HLJDD group there had no significant difference. It suggested HLJDD had potential to promote apoptosis, the effect of 2.5 mg/mL HLJDD was similar with that of paclitaxel.

### HLJDD inhibited G1 phase and arrested in S phase on cell division cycle

From above results, we confirmed that HLJDD inhibited the growth of OSCC and promoted the cell apoptosis. It was still unclear whether it had an influence on cell cycle. Based on network pharmacological enrichment analysis results (cell cycle arrest; cell proliferation, etc.) It was necessary to verify the effect of HLJDD on cell cycle to provide evidence to explain the effect of HLJDD on cell proliferation.

**Fig. 6:**
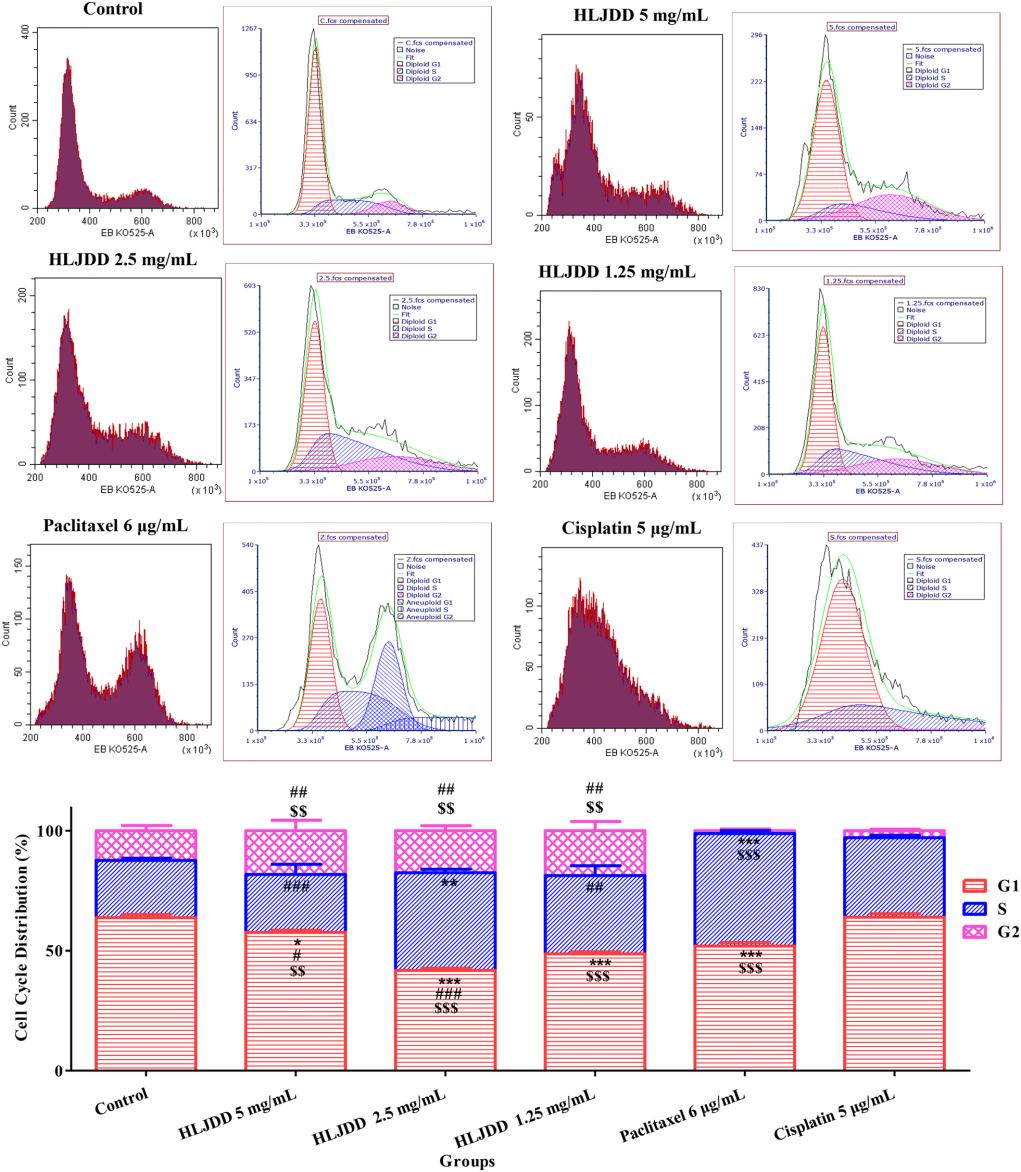
The effects of HLJDD (HLJDD 5 mg/mL, HLJDD 2.5 mg/mL, HLJDD 1.25 mg/mL), paclitaxel 6 μg/mL and cisplatin 5 μg/mL on cell cycle. Cell cycle histograms determined by CytoFLEX LX flow cytometry analyzer following Ethidium Bromide (EB) staining. SCC-25 cells were treated with HLJDD 5 mg/mL, HLJDD 2.5 mg/mL, HLJDD 1.25 mg/mL, paclitaxel 6 μg/mL and cisplatin 5 μg/mL for 24 h. *** *p* < 0.001, **p < 0.01, * *p* < 0.05 *vs*. control group. ### *p* < 0.001, ## *p* < 0.01, # *p* < 0.05 *vs*. paclitaxel group. $$$ *p* < 0.001, $$ *p* < 0.01 vs cisplatin group. n = 3

In Fig. 6, compared with control group, HLJDD (1.25 mg/mL, 2.5 mg/mL, 5 mg/mL) and 6 μg/mL paclitaxel significantly reduced G1 phase (DNA pre-synthesis phase), especially 2.5 mg/mL HLJDD. Moreover, 2.5 mg/mL HLJDD and paclitaxel were significantly elevated the percentages S phase (DNA synthesis phase). It revealed that 2.5 mg/mL HLJDD promoted S phase arrest rather than 1.25 mg/mL and 5 mg/mL HLJDD. Altogether, 2.5 mg/mL HLJDD had more similar effect to paclitaxel, which reduced proportion of G1 phase and increased proportion of S phase. All above, 2.5 mg/mL HLJDD interfered with cell cycle by DNA synthesis inhibition, S phase arrest and consequently suppressed cell proliferation. It was consistent with the biological process enrichment analysis result of network pharmacology.

### HLJDD inhibited the migration of OSCC

**Fig. 7:**
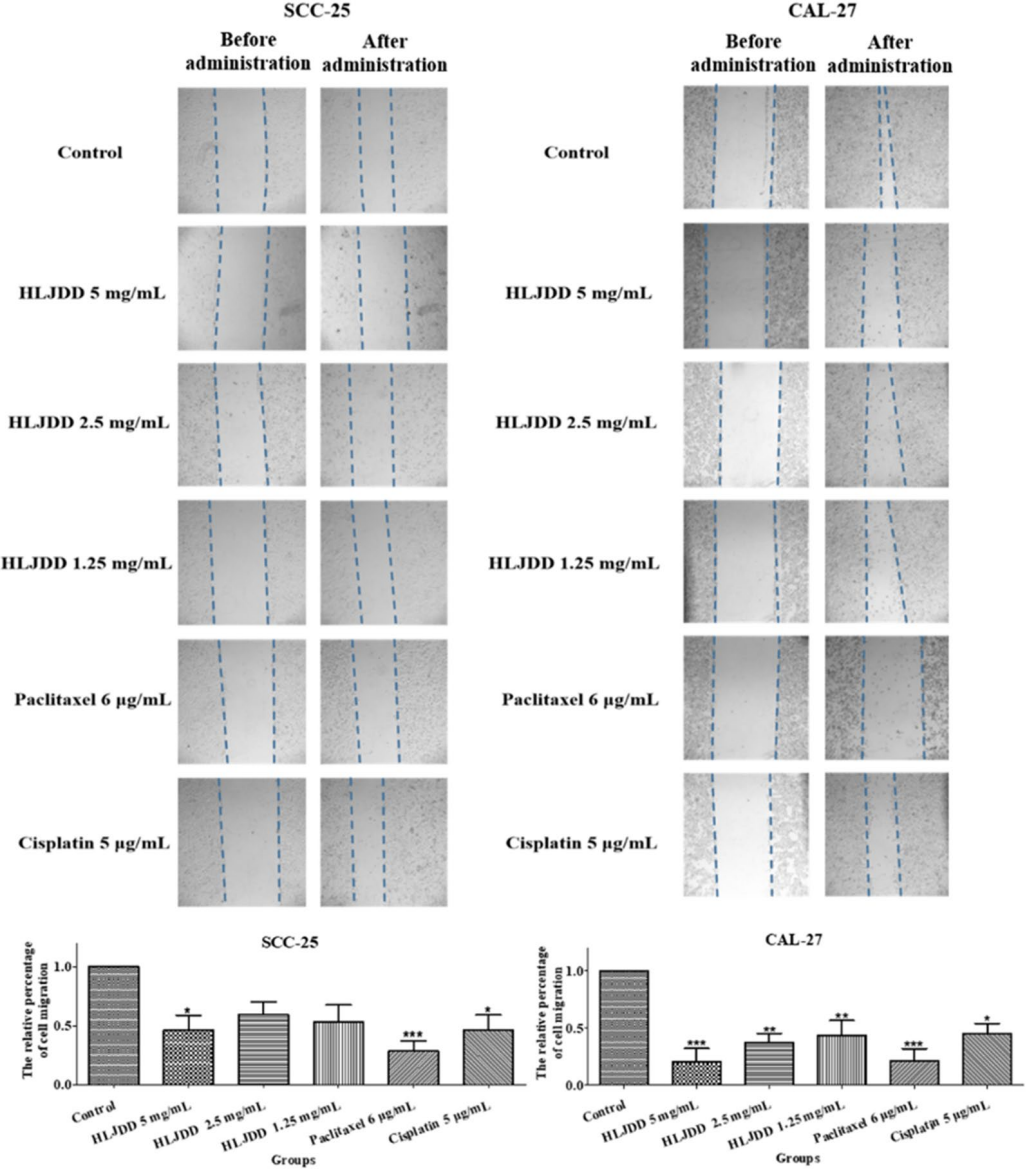
The representative images of wound-healing assay in six groups. SCC-25 cells (left part) and CAL-27 cells (right part) were treated with HLJDD 5 mg/mL, HLJDD 2.5 mg/mL, HLJDD 1.25 mg/mL, paclitaxel 6 μg/mL and cisplatin 5 μg/mL for 24 h, respectively. All images both before administration and after administration were taken under microscope (original magnification, ×40). The blue dotted line represented the area scratched by the 1000 µL-pipette tips. The statistics were expressed as Mean ± SEM, n = 7 (SCC-25 cells). * *p* < 0.05 *vs.* control group. The statistics were expressed as Mean ± SEM, n = 4 (CAL-27 cells). *** *p* < 0.001, **p < 0.01, * *p* < 0.05 vs*.* control group

We performed wound healing assay to illustrate the migration of cells. In Fig. 7, the inhibitory effects of HLJDD on CAL-27 cell line were in dose-dependent manner and more obvious than that on SCC-25 cell line. On two cells, the inhibitory effects of 1.25 mg/mL HLJDD and cisplatin were similar, while 5 mg/mL HLJDD and paclitaxel presented stronger inhibitory effect than others. Stated thus, HLJDD significantly suppressed the migration of OSCC.

### HLJDD inhibited phosphorylation of ERK1/2 and NF-κB p65 (S468)

**Fig. 8:**
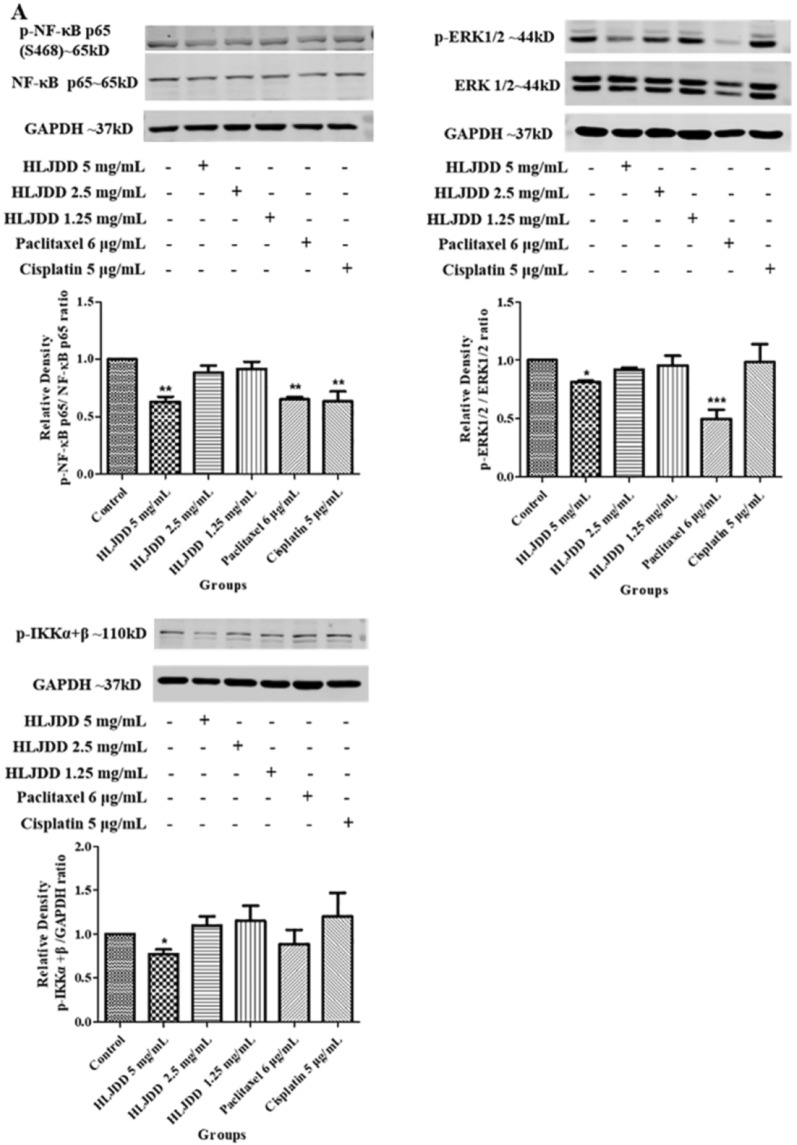

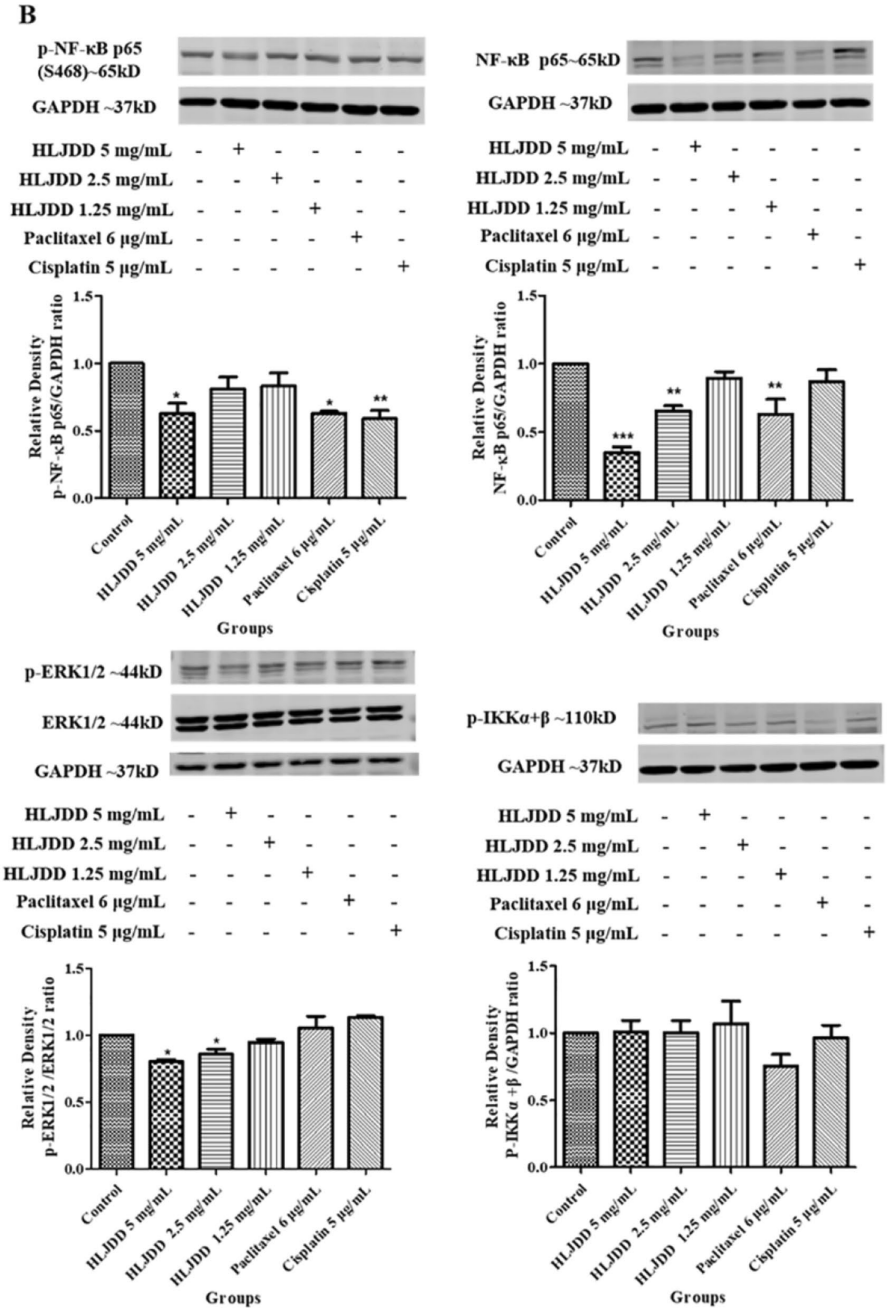
HLJDD inhibited NF-κB and ERK pathway on CAL-27 and SCC-25. **A** CAL-27 cells were treated with HLJDD 5 mg/mL, HLJDD 2.5 mg/mL, HLJDD 1.25 mg/mL, paclitaxel 6 μg/mL and cisplatin 5 μg/mL for 24 h. We detected the expression of p-NF-κB p65 (S468), NF-κB p65, p-ERK1/2 and p-IKK α + β by using the western blot analysis. The statistics were expressed as Mean ± SEM, n = 3. *** *p* < 0.001, **p < 0.01, * *p* < 0.05 *vs.* control group. **B** SCC-25cells were treated with HLJDD 5 mg/mL, HLJDD 2.5 mg/mL, HLJDD 1.25 mg/mL, paclitaxel 6 μg/mL and cisplatin 5 μg/mL for 24 h. We detected the expression of p-NF-κB p65 (S468), NF-κB p65, p-ERK1/2 and p-IKK α + β by using the western blot analysis. The statistics were expressed as Mean ± SEM, n = 3. *** *p* < 0.001, **p < 0.01, * *p* < 0.05 vs. control group

HLJDD inhibited the late stage of apoptosis and suppressed the cell cycle by arresting S phase. Meanwhile, HLJDD inhibited the migration of OSCC but the mechanism was still unknown. The results of network pharmacological enrichment analysis provided directions for the exploration of these biological processes. Then we focused on the TOP30 of KEGG pathway enrichment results. HLJDD reduced the phosphorylation of ERK1/2 in dose-dependent manners on both SCC-25 and CAL-27 cell lines (Fig. 8A and B). Additionally, 5 mg/ mL HLJDD showed nearly 40% inhibitory rate on the phosphorylation of NF-κB p65 (S468) both SCC-25 and CAL-27 cell lines. Interestingly, on SCC-25 cell line, p-NF-κB p65 reduced in a dose-dependent manner, which did not find on CAL-27 cell line. Moreover, we found 5 mg/ mL HLJDD inhibited the phosphorylation of IKK α + β on CAL-27 not SCC-25. At the same time, we checked other phosphorylation sites of NF-κB p65, including S529 and S276, but failed to find the significant change (Additional file 1: Fig. S1 A and B), suggested that HLJDD specifically inhibited phosphorylation of S468 site. It was obvious that the paclitaxel had a higher inhibitory effect on CAL-27 and SCC-25 cell lines than that of HLJDD, but the inhibitory effect of 5 mg/mL HLJDD on phosphorylation was almost same like paclitaxel. Altogether, HLJDD might inhibit p-ERK1/2 and p-NF-κB p65 (S468) to play a therapeutic role on OSCC.

## Discussion

In our study, we explored the Huanglianjiedu Decoction (HLJDD) potential mechanism on oral squamous cell carcinoma (OSCC) based on network pharmacology and discovered the antitumor effect of HLJDD on OSCC cell lines. According to the enrichment analysis of core targets by Gene Ontology (GO) (Biological Process (BP), Cellular Component (CC) and Molecular Function (MF)) and Kyoto Encyclopedia of Genes and Genomes (KEGG) analysis, we found the effects of HLJDD on OSCC involved in cell proliferation, apoptosis and cell cycle. Additionally, we found several relative signaling pathways including mitogen-activated protein kinase/extracellular regulated protein kinases (MAPK/ERK) pathways, nuclear factor-kappa B (NF-κB) pathway and so on. Further in vitro experiments, we confirmed that HLJDD inhibited cell proliferation on CAL-27 and SCC-25 cell by promoting the late apoptosis, inhibiting the G1 phase (pre-DNA synthesis) and arresting the S phase in the cell cycle. HLJDD significantly inhibited phosphate-extracellular regulated protein kinases1/2 (p-ERK1/2), p-NF-κB p65 (S468). These results suggest that HLJDD could play a therapeutic role in OSCC via inhibiting p-ERK1/2 and p-NF-κB p65 (S468).

Network pharmacology develops rapidly on basis of omics and large databases which provide the basic data to investigators for further analysis. In addition, the prediction of drug targets is another highlight of network pharmacology [40]. More and more studies used network pharmacology to investigate the effect of traditional Chinese medicine (TCM) on diseases. Based on pharmacokinetics datasets, prediction and screening by network pharmacology, Yue et al. successfully applied the molecular network to explain the mechanism of Angelica safflower on blood stasis syndrome [41]; Wang et al. used bioinformatics and network pharmacology to clarify the active ingredients, targets and pathways of Tianfoshen oral solution effectively worked on colorectal cancer [42]; Wu et al. applied databases, bioinformatics and network pharmacology to establish PPI networks and screened out the targets of curcumin on interstitial cystitis [43]. But network pharmacology also had disadvantages, such as its uncertainty and too wide coverage, which require more experiments to verify its accuracy. Even some items don't quite fit for human species.

The GO and KEGG pathway enrichment analysis of core targets provided a direction to explore the antitumor effect and mechanism of HLJDD on OSCC. The KEGG enrichment analysis was consistent with GO analysis about cell cycle and apoptosis. Besides, we focused on some pathways of TOP30. Firstly, we verified PI3K-AKT which had high Rich Factor. But the expression of p-AKT and AKT1/2/3 did not change after administration detected by western blot. It suggested the effect of HLJDD may not involve in PI3K-AKT pathway (Additional file 1: Fig. S1A and B). According to GO-BP and GO-CC enrichment results, HLJDD may involve in the activation of MAPK, kinase activity and binding. Therefore, we detected the expression of MAPK family (p38 and JNK) and found that the expression of p-p38 was increased after administration on both SCC-25 and CAL-27, while the expression of JNK showed no band detected by western blot on CAL-27 (the reason may relate to the administration for 24 h) (Fig. 8, Additional file 1: Fig. S1 A and B). Wu et al. verified the anticancer properties of pristimerin on SCC-25 and CAL-27, which are related to the inhibition of MAPK/ ERK1/2 and AKT pathway [44]. The reason we did not get similar results about AKT may relate to the different stimuli and the experimental conditions. In our validation experiments, some results were not completely consistent on the two types of OSCC cell lines SCC-25 and CAL-27, for example, paclitaxel significantly inhibited p-ERK1/2 in CAL-27, but not in SCC-25. We considered the possible reasons maybe different cell lines have different sensitivity for the drugs.

Surprisingly, after administration of HLJDD on SCC-25 the expression of NF-κB p65 was reduced. We speculated that the synthesis of NF-κB p65 was decreased or degradation was increased. Sergej Skvortsov et al. found that CTFB, a novel anticancer drug, its anti-tumor activity was associated with down-regulation of NF-κB p65 expression by activation of proteasome in SCC-25 and CAL-27 cells [45]. Whether NF-κB p65 down regulation by HLJDD is related to proteasome activation or not needs further verification.

At first, we chose HLJDD (40 mg/mL, 20 mg/mL, 10 mg/mL, 5 mg/mL, 2.5 mg/mL and 1.25 mg/mL), paclitaxel (6 μg/mL) and cisplatin (5 μg/mL) to stimulate CAL-27, we found that prolonging incubated time, the number of cells decreased visually. It indicated that HLJDD, paclitaxel and cisplatin inhibit cells proliferation (Additional file 2: Fig. S2). Combining with the CCK-8 results (Additional file 3: Fig. S3 A) and SRB results (Additional file 3: Fig. S3 B), it was obvious to see that the best time for cell harvesting was 24 h after administration, because the cell inhibitory rate was greater than 60-70% at 48 h or 72 h after administration. The SRB results showed that 5 mg/mL HLJDD group had higher inhibitory effect than that of 10 mg/mL HLJDD group after administration for 24 h (Additional file 3: Fig. S3 B). That’s why we chose doses of 1.25 mg/mL, 2.5 mg/mL, and 5 mg/mL HLJDD. However, HLJDD did not showed obvious dose–effect relationship. 5 mg/mL HLJDD showed stronger inhibition on cell migration (Fig. 7), and significantly reduced p-ERK1/2 and p-NF-κB p65 (S468) expression (Fig. 8), while 2.5 mg / mL HLJDD showed stronger suppression on cell cycle and apoptosis (Figs. 5 and 6). So, we speculated that 2.5 mg/mL HLJDD was a relatively sensitive concentration for cell cycle and apoptosis, while 5 mg/mL HLJDD may have stronger action on other biological processes of cell. Thus, in different evaluation indicators, different concentrations reflect different results. As for the observations on late stage apoptosis (Additional file 4: Fig. S4) and cell division cycle (Additional file 5: Fig. S5) on CAL-27, 1.25 mg/mL HLJDD significantly increased apoptosis in late stage, while 2.5 mg/mL and 5 mg/mL HLJDD just slightly increased. The ability of 1.25 mg/mL HLJDD and 5 μg/mL cisplatin to induce late apoptosis on CAL-27 were similar, which were consistent with the results of SCC-25 (Fig. 5). But on CAL-27, the promoting effect of 2.5 mg/mL HLJDD on late apoptosis was not as obvious as that on SCC-25. Although the effects of the same dose of HLJDD were inconsistent on SCC-25 and CAL-27, all three doses of HLJDD can induce late stage apoptosis in SCC-25 and CAL-27. In Fig. S5, the percentage of cell in S phase in HLJDD (5 mg/mL, 2.5 mg/mL, 1.25mg/mL) groups were 31.44%, 34.48%, 32.53%, respectively, while that of control, paclitaxel (6 μg/mL) and cisplatin (5 μg/mL) groups were 24.40%, 24.10% and 57.19%, respectively. HLJDD and cisplatin could elevate the percentages of cell in S phase, but only cisplatin group had a significant increase when compared with control group. In a word, HJLDD can promote late stage of cell apoptosis and increase the proportion of S phase in the cell cycle to inhibit the proliferation of SCC-25 and CAL-27 cell lines.

Based on our results, we found that not all enrichment analysis results were in line with experiments. It indicated that network pharmacology just provided some directions and specific targets needed further experimental verification.

## Conclusions

Our study was the first time using the network pharmacology to explore the mechanism about HLJDD on OSCC. We screened 52 core targets by topological analysis. Based on GO-BP, GO-CC, GO-MF and KEGG pathway enrichment analysis, we found that 52 core targets were involved in biological processes such as cell cycle, apoptosis and activation of MAPK pathway. We further experimentally verified on OSCC cell lines (SCC-25 and CAL-27). The results supported that HLJDD inhibited cell proliferation on CAL-27 and SCC-25 by promoting the late apoptosis of the cells, inhibiting the G1 phase (pre-DNA synthesis), arresting the S phase in the cell cycle on SCC-25 and inhibiting p-ERK1 / 2 and p-NF-κB p65 (S468) on SCC-25 and CAL-27. So HLJDD could be a potential drug for the treatment of OSCC.

## Supplementary Information

**Additional file 1:** **Fig. S1.** Study on mechanism of HLJDD on CAL-27, SCC-25 cell lines and quantitative graphs. (A) CAL-27 cells were treated with HLJDD 5 mg/mL, HLJDD 2.5mg/mL, HLJDD 1.25 mg/mL, paclitaxel 6 μg/mL and cisplatin 5μg/mL for 24 hours. We detected the expression of p-AKT, AKT, p-p38, p-NF-κB p65 (S529) and p-NF-κB p65 (S276) by using the western blot analysis. n = 2–3. (B) SCC-25 cells were treated with HLJDD 5 mg/mL, HLJDD 2.5 mg/mL, HLJDD 1.25 mg/mL, paclitaxel 6 μg/mL and cisplatin 5 μg/mL for 24 hours. We detected the expression of p-AKT, AKT, p-p38, p-JNK and p-NF-κB p65 (S276) by using the western blot analysis. n = 2–3.

**Additional file 2:** **Fig. S2.** Representative images of six groups (original magnification 200×). CAL-27 cells were treated with HLJDD (40 mg/mL, 20 mg/mL, 10 mg/mL, 5 mg/mL, 2.5 mg/mL and 1.25 mg/mL), paclitaxel 6 μg/mL and cisplatin 5 μg/mL.

**Additional file 3: Fig. S3.** The inhibitory effects of HLJDD, paclitaxel and cisplatin on CAL-27 cell line were detected by Sulforhodamine B (SRB) (KeyGEN BioTECH, Nanjing, China) and CCK-8 measurement (C0037, Beyotime Biotechnology., Shanghai, China) after administration for 24, 48 and 72 h. n=6. (A) CAL-27 cells were treated with HLJDD (40 mg/mL, 20 mg/mL, 10 mg/mL, 5 mg/mL, 2.5 mg/mL and 1.25 mg/mL), paclitaxel 6 μg/mL and cisplatin 5 μg/mL. After administration for 24, 48 and 72 h, we used CCK-8 measurement to analyze the inhibition effect of drugs on CAL-27 cell line. (B) CAL-27 cells were treated with HLJDD (10 mg/mL, 5 mg/mL, 2.5 mg/mL and 1.25 mg/mL), paclitaxel 6 μg/mL and cisplatin 5 μg/mL. After administration for 24, 48 and 72 h, we used SRB measurement to analyze the inhibition effect of drugs on CAL-27 cell line.

**Additional file 4: Fig. S4.** The representative contour plots of six groups and the quantitative statistical chart. CAL-27 cells were treated with HLJDD 5 mg/mL, HLJDD 2.5 mg/mL, HLJDD 1.25 mg/mL, paclitaxel 6 μg/mL and cisplatin 5 μg/mL for 24 h. Late stage of apoptosis determined by CytoFLEX S cytometry analyzer (Beckman Coulter, Inc. 250S. Kraemer Boulevard Brea, CA 92821, USA) following Annexin V APC and 7-AAD staining (abs50008, absin Bioscience Inc., Shanghai, China). Both Annexin V APC and 7-AAD positive cells represented that the cells were in late stage of apoptosis. The statistics were expressed as Mean ± SEM, n=3. *** *p* < 0.001, ** *p* < 0.01 vs. control group. ### *p* < 0.001, ## *p* < 0.01 vs. paclitaxel group. There were no significant differences between other groups and cisplatin group.

**Additional file 5: Fig. S5.** The effects of HLJDD (5 mg/mL, 2.5 mg/mL, 1.25 mg/mL), paclitaxel 6 μg/mL and cisplatin 5 μg/mL on cell cycle of CAL-27 cell line. Cell cycle histograms determined by CytoFLEX S flow cytometry analyzer (Beckman Coulter, Inc. 250S. Kraemer Boulevard Brea, CA 92821, USA) following Propidium Iodide (PI) staining measurement (C1052, Beyotime Biotechnology., Shanghai, China). CAL-27 cells were treated with HLJDD 5 mg/mL, HLJDD 2.5 mg/mL, HLJDD 1.25 mg/mL, paclitaxel 6 μg/mL and cisplatin 5 μg/mL for 24 h. *** *p* < 0.001 vs. control group. ### *p* < 0.001 vs. paclitaxel group. $$$ *p *< 0.001, $$ *p *< 0.01 vs. cisplatin group, n=3.

## Data Availability

The data sets used and/or analyzed during the current study are available from the corresponding author on reasonable request.

## Abbreviations

AKT: Protein kinase B
GO: Gene Ontology
HLJDD: Huanglianjiedu Decoction
HPLC: High Performance Liquid Chromatography
KEGG: Kyoto Encyclopedia of Genes and Genomes
MAPK: Mitogen-activated protein kinase
MS: Mass spectrometry
NF-κB: Nuclear factor-kappa B
OMIM: Online Mendelian Inheritance in Man
OSCC: Oral squamous cell carcinoma
p-ERK1/2: Phosphate-extracellular regulated protein kinases1/2
PPI: Protein–protein interaction
TCMSP: Traditional Chinese Medicine Systems Pharmacology Database and Analysis Platform
TTD: Therapeutic Target Database
