# Supplementary figures and images for: Huanglianjiedu Decoction as an effective treatment for oral squamous cell carcinoma based on network pharmacology and experimental validation
Source: Cancer Cell Int. 2021 Oct 21;21:553. doi: 10.1186/s12935-021-02201-6 (PMC8529748; doi:10.1186/s12935-021-02201-6)

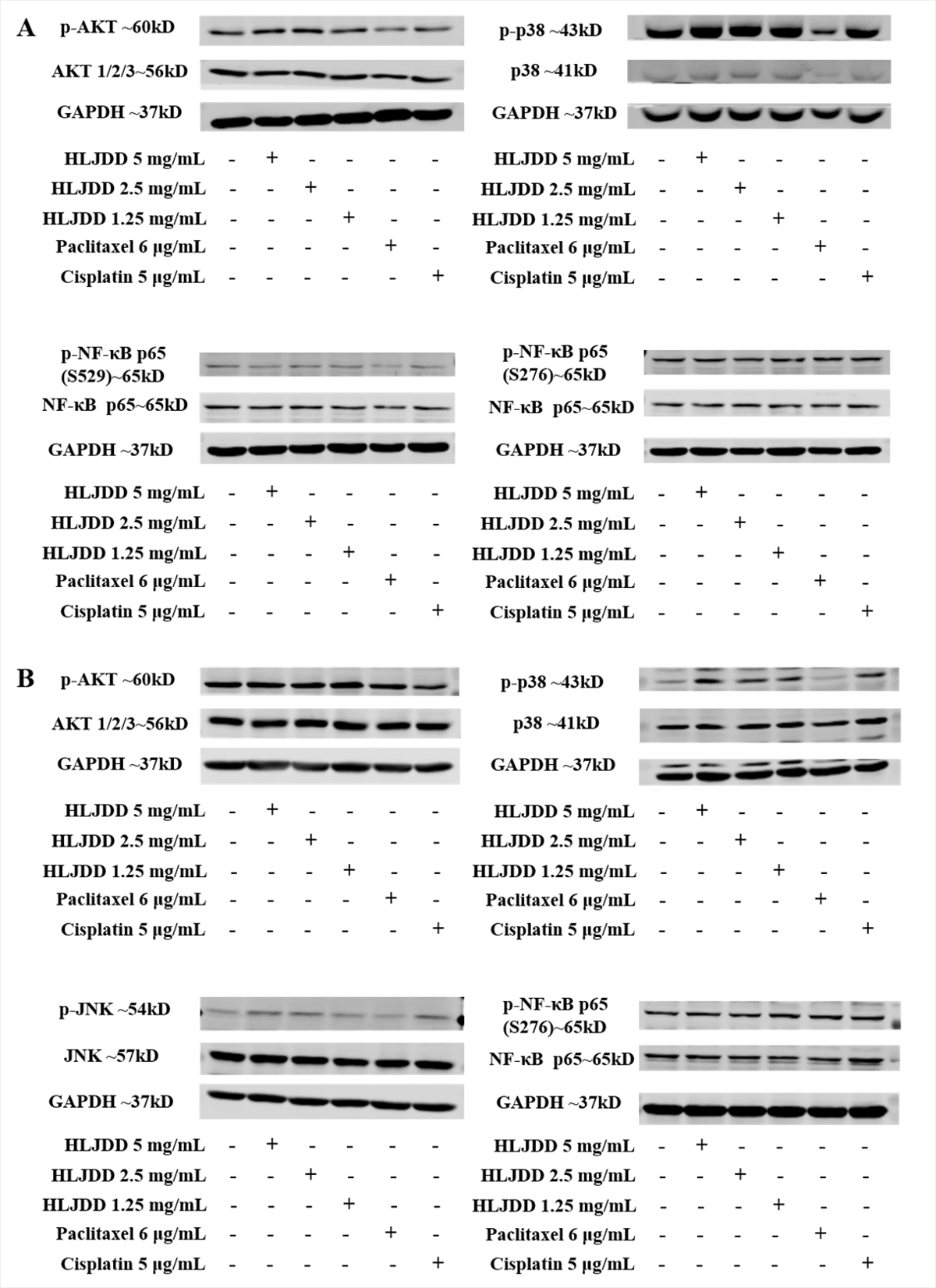

Supplement: Supplementary file 1 — Additional file 1: Fig. S1. Study on mechanism of HLJDD on CAL-27, SCC-25 cell lines and quantitative graphs. (A) CAL-27 cells were treated with HLJDD 5 mg/mL, HLJDD 2.5mg/mL, HLJDD 1.25 mg/mL, paclitaxel 6 μg/mL and cisplatin 5μg/mL for 24 hours. We detected the expression of p-AKT, AKT, p-p38, p-NF-κB p65 (S529) and p-NF-κB p65 (S276) by using the western blot analysis. n = 2–3. (B) SCC-25 cells were treated with HLJDD 5 mg/mL, HLJDD 2.5 mg/mL, HLJDD 1.25 mg/mL, paclitaxel 6 μg/mL and cisplatin 5 μg/mL for 24 hours. We detected the expression of p-AKT, AKT, p-p38, p-JNK and p-NF-κB p65 (S276) by using the western blot analysis. n = 2–3. [file 12935_2021_2201_MOESM1_ESM.tif]

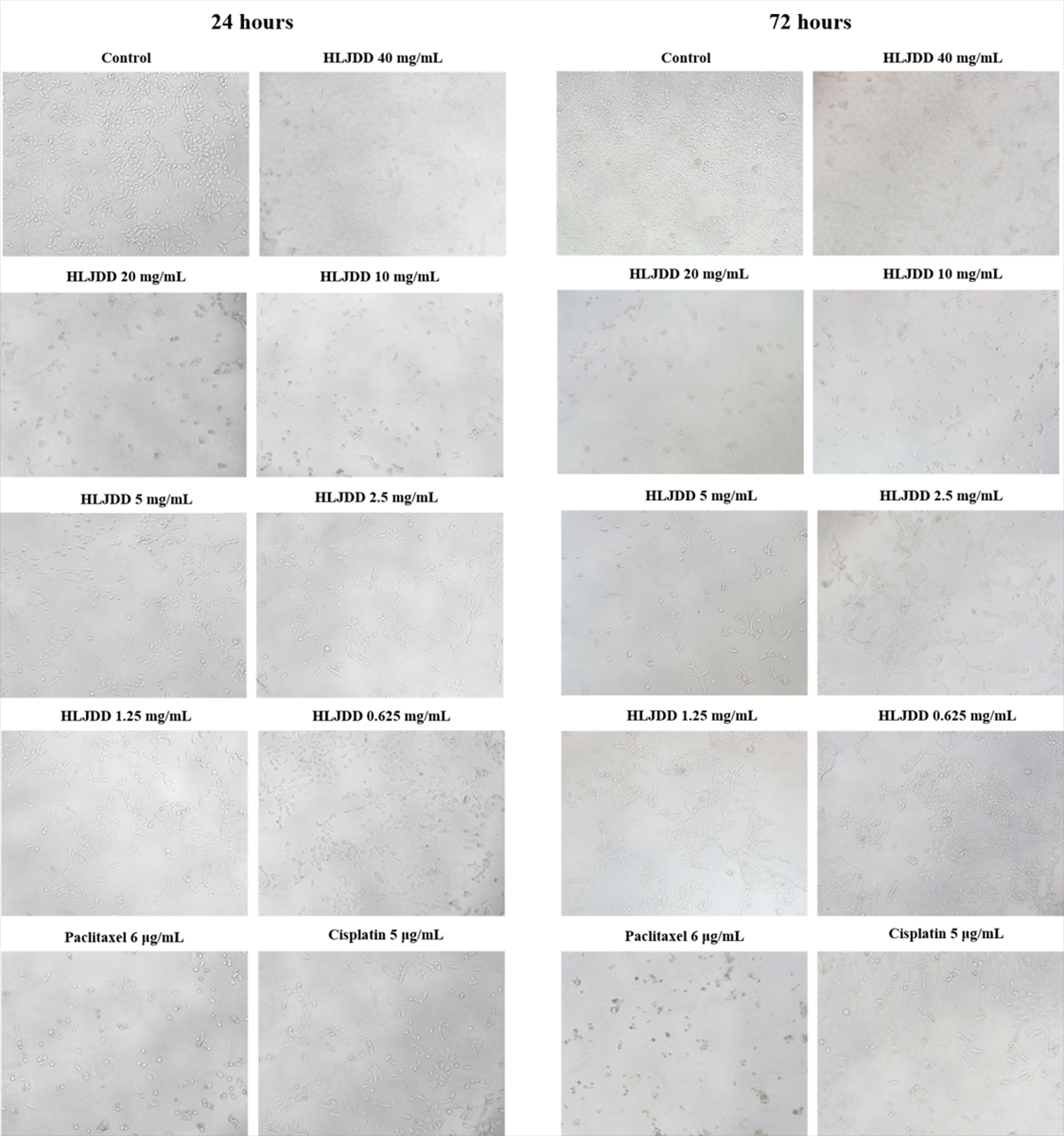

Supplement: Supplementary file 2 — Additional file 2: Fig. S2. Representative images of six groups (original magnification 200×). CAL-27 cells were treated with HLJDD (40 mg/mL, 20 mg/mL, 10 mg/mL, 5 mg/mL, 2.5 mg/mL and 1.25 mg/mL), paclitaxel 6 μg/mL and cisplatin 5 μg/mL. [file 12935_2021_2201_MOESM2_ESM.tif]

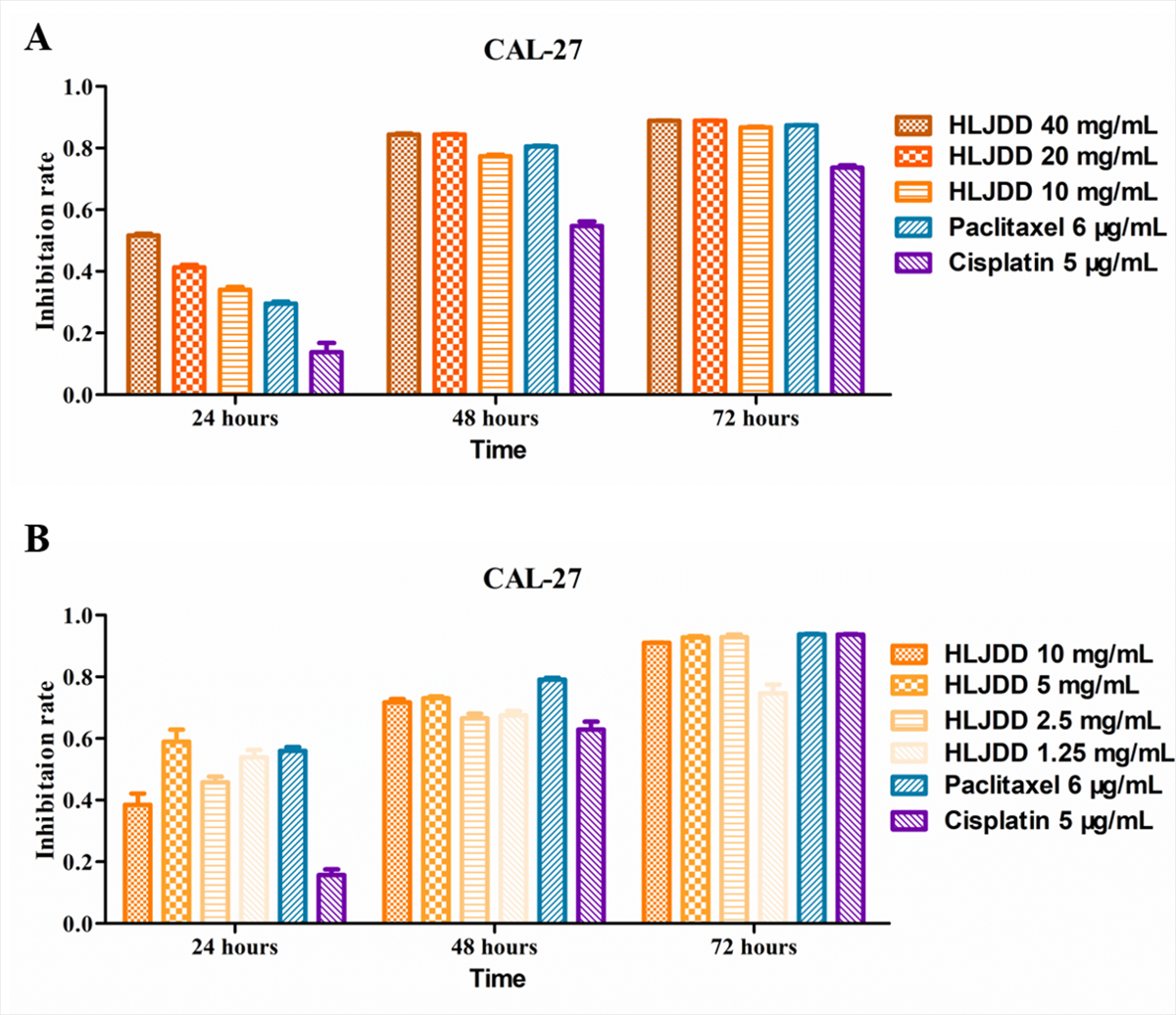

Supplement: Supplementary file 3 — Additional file 3: Fig. S3. The inhibitory effects of HLJDD, paclitaxel and cisplatin on CAL-27 cell line were detected by Sulforhodamine B (SRB) (KeyGEN BioTECH, Nanjing, China) and CCK-8 measurement (C0037, Beyotime Biotechnology., Shanghai, China) after administration for 24, 48 and 72 h. n=6. (A) CAL-27 cells were treated with HLJDD (40 mg/mL, 20 mg/mL, 10 mg/mL, 5 mg/mL, 2.5 mg/mL and 1.25 mg/mL), paclitaxel 6 μg/mL and cisplatin 5 μg/mL. After administration for 24, 48 and 72 h, we used CCK-8 measurement to analyze the inhibition effect of drugs on CAL-27 cell line. (B) CAL-27 cells were treated with HLJDD (10 mg/mL, 5 mg/mL, 2.5 mg/mL and 1.25 mg/mL), paclitaxel 6 μg/mL and cisplatin 5 μg/mL. After administration for 24, 48 and 72 h, we used SRB measurement to analyze the inhibition effect of drugs on CAL-27 cell line. [file 12935_2021_2201_MOESM3_ESM.tif]

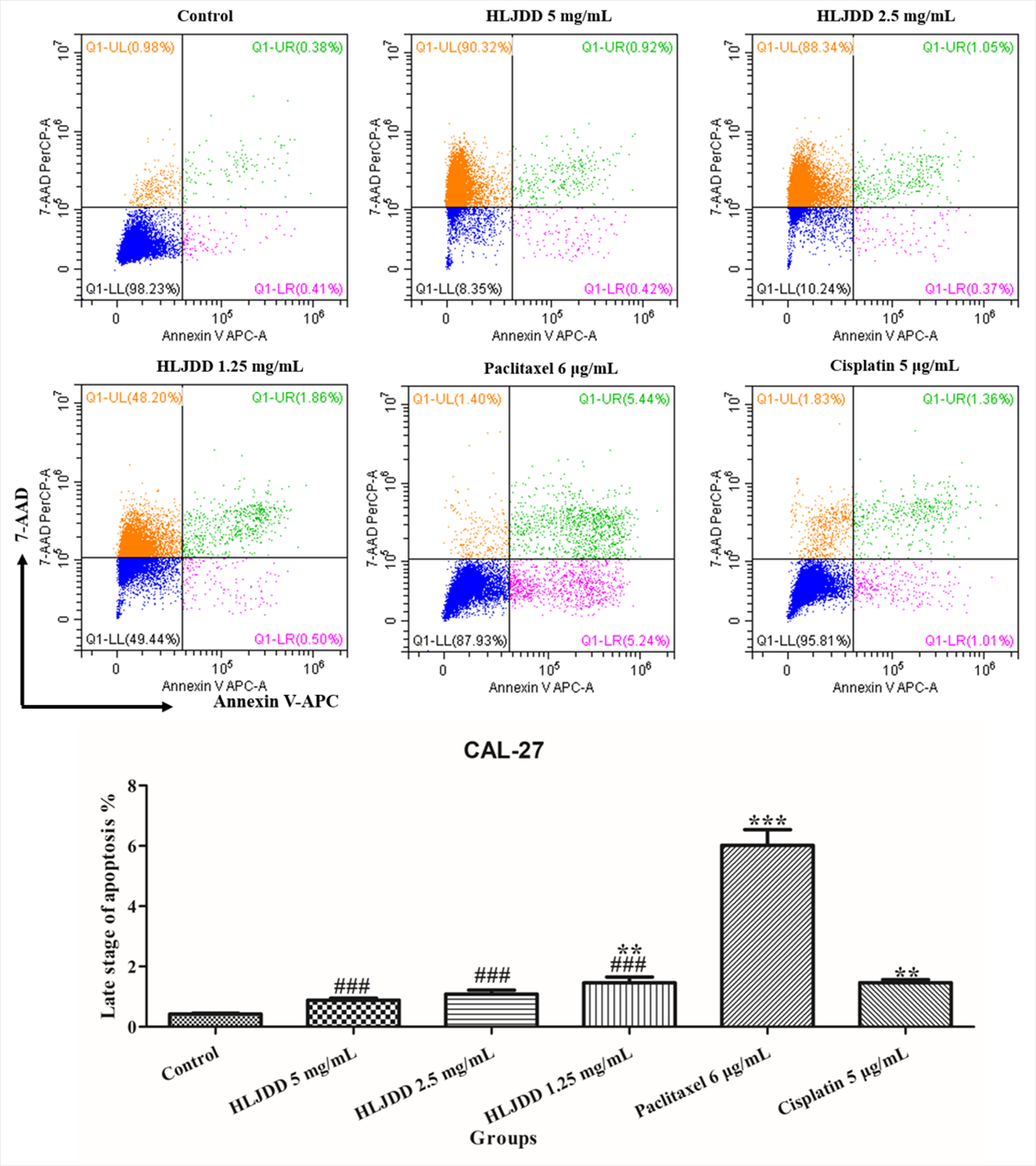

Supplement: Supplementary file 4 — Additional file 4: Fig. S4. The representative contour plots of six groups and the quantitative statistical chart. CAL-27 cells were treated with HLJDD 5 mg/mL, HLJDD 2.5 mg/mL, HLJDD 1.25 mg/mL, paclitaxel 6 μg/mL and cisplatin 5 μg/mL for 24 h. Late stage of apoptosis determined by CytoFLEX S cytometry analyzer (Beckman Coulter, Inc. 250S. Kraemer Boulevard Brea, CA 92821, USA) following Annexin V APC and 7-AAD staining (abs50008, absin Bioscience Inc., Shanghai, China). Both Annexin V APC and 7-AAD positive cells represented that the cells were in late stage of apoptosis. The statistics were expressed as Mean ± SEM, n=3. *** p < 0.001, ** p < 0.01 vs. control group. ### p < 0.001, ## p < 0.01 vs. paclitaxel group. There were no significant differences between other groups and cisplatin group. [file 12935_2021_2201_MOESM4_ESM.tif]

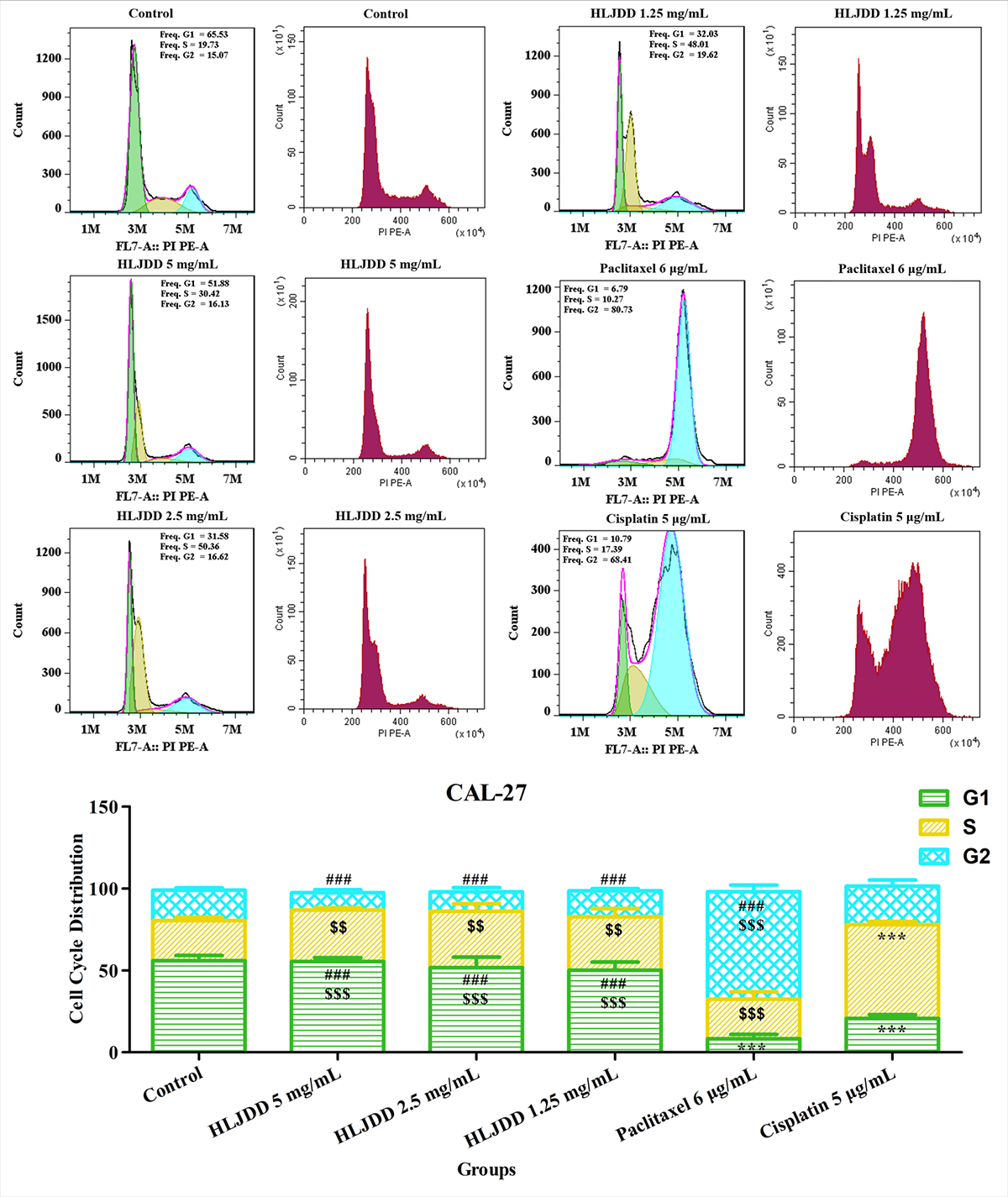

Supplement: Supplementary file 5 — Additional file 5: Fig. S5. The effects of HLJDD (5 mg/mL, 2.5 mg/mL, 1.25 mg/mL), paclitaxel 6 μg/mL and cisplatin 5 μg/mL on cell cycle of CAL-27 cell line. Cell cycle histograms determined by CytoFLEX S flow cytometry analyzer (Beckman Coulter, Inc. 250S. Kraemer Boulevard Brea, CA 92821, USA) following Propidium Iodide (PI) staining measurement (C1052, Beyotime Biotechnology., Shanghai, China). CAL-27 cells were treated with HLJDD 5 mg/mL, HLJDD 2.5 mg/mL, HLJDD 1.25 mg/mL, paclitaxel 6 μg/mL and cisplatin 5 μg/mL for 24 h. *** p < 0.001 vs. control group. ### p < 0.001 vs. paclitaxel group. $$$ p < 0.001, $$ p < 0.01 vs. cisplatin group, n=3. [file 12935_2021_2201_MOESM5_ESM.tif]
